# Non-biogroup 1 or 2 Strains of the Emerging Zoonotic Pathogen *Escherichia albertii*, Their Proposed Assignment to Biogroup 3, and Their Commonly Detected Characteristics

**DOI:** 10.3389/fmicb.2019.01543

**Published:** 2019-07-05

**Authors:** Koichi Murakami, Eriko Maeda-Mitani, Hirokazu Kimura, Mikiko Honda, Tetsuya Ikeda, Wakana Sugitani, Takayuki Konno, Kimiko Kawano, Yoshiki Etoh, Nobuyuki Sera, Fuminori Mizukoshi, Takehito Saitoh, Yoshiaki Kawamura, Taisei Ishioka, Makoto Ohnishi, Kazunori Oishi, Shuji Fujimoto

**Affiliations:** ^1^Infectious Disease Surveillance Center, National Institute of Infectious Diseases, Musashimurayama, Japan; ^2^Fukuoka Institute of Health and Environmental Sciences, Dazaifu, Japan; ^3^School of Medical Technology, Faculty of Health Science, Gunma Paz University, Takasaki, Japan; ^4^Fukuoka City Institute for Hygiene and the Environment, Fukuoka, Japan; ^5^Hokkaido Institute of Public Health, Sapporo, Japan; ^6^Kumamoto City Environmental Research Institute, Kumamoto, Japan; ^7^Akita Prefectural Research Center for Public Health and Environment, Akita, Japan; ^8^Miyazaki Prefectural Institute for Public Health and Environment, Miyazaki, Japan; ^9^Tochigi Prefectural Institute of Public Health and Environmental Science, Utsunomiya, Japan; ^10^Department of Microbiology, School of Pharmacy, Aichi Gakuin University, Nagoya, Japan; ^11^Takasaki City Health Center, Takasaki, Japan; ^12^Department of Bacteriology I, National Institute of Infectious Diseases, Tokyo, Japan; ^13^Professor Emeritus, Kyushu University, Fukuoka, Japan

**Keywords:** biogroups, *Escherichia albertii*, food microbiology, identification, public health

## Abstract

*Escherichia albertii*, a zoonotic enteropathogen, is responsible for outbreaks of disease in humans. Identifying strains of *E. albertii* by phenotypic characterization tests is difficult because of its poorly defined properties. Screening its phenotypic characteristics is, nevertheless, a necessary prerequisite for further genetic analysis of its properties, and species-specific polymerase chain reaction (PCR) analysis can be used to type the pathogen. While two *E. albertii* biogroups (1 and 2) have been described, strains with characteristics divergent from both biogroups have been reported worldwide. The aim of the present study was to evaluate the characteristics of non-biogroup 1 or 2 strains, and discern the characteristics common to all of the *E. albertii* strains from this study. Altogether, 107/414 field isolates were selected for examination based on pulsed-field gel electrophoresis analysis. The 107 strains were isolated from 92 sources, including humans and pigeon feces, other wild birds, and retail chicken livers. All strains were then examined using various culture-based, biochemical (API 50CHE tests, API Zym test, and others) and molecular (virulence gene screening, multi-locus sequence analysis) testing methods. Our results revealed that all field strains (*n* = 107) showed non-biogroup 1 or 2 characteristics, with multiple sequence differences. Variations in indole production and the lysine decarboxylase activity profiles among the isolates made identification of *E. albertii* very difficult. Therefore, we propose that non-biogroup 1 or 2 of *E. albertii* should be assigned to biogroup 3 to make screening of them easier in public health and clinical laboratory settings. Clearly, having group criteria for indole-negative/lysine-positive, indole-positive/lysine-negative, and indole-positive/lysine-positive *E. albertii* biogroups 1, 2, and 3 strains, respectively, should provide for more accurate identification of *E. albertii* isolates. Based on our findings, we recommend that isolates displaying phenotype mobility-negativity (sulfide-indole-motility medium, 37°C), hydrogen sulfide production-negativity (triple sugar iron medium), acid production-negativity from xylose, negative β-glucuronidase activity properties, and showing indole production and lysine decarboxylase activity profiles in accordance with one of the three biogroups, should be further assessed using an *E. albertii*-specific PCR assay.

## Introduction

Phenotypic identification of the zoonotic human enteropathogen *Escherichia albertii* remains difficult despite it being recognized as a species since 2003 (Huys et al., 2003). *E. albertii* is mainly isolated from domestic and wild birds (Oaks et al., 2010), along with humans (Ooka et al., 2012), and human disease outbreaks caused by it have been reported (Ooka et al., 2013). A lack of information on the prevalence of *E. albertii*, as well as difficulties with its isolation and identification have hampered attempts to define the routes involved in its zoonotic transmission to humans (Nimri, 2013; Maeda et al., 2015). At present, accurate identification of *E. albertii* is restricted to genotyping techniques such as polymerase chain reaction (PCR) (Maeda et al., 2014), because some *E. albertii* strains were initially misidentified as *Escherichia coli* based on unreliable phenotyping methods (Murakami et al., 2014b). We have previously recommended that non-motile, *eae* positive, D-xylose and L-rhamnose fermentation negative isolates should be screened to determine whether or not they are *E. albertii* (Murakami et al., 2014b). Public health and clinical laboratories worldwide should also document the common phenotypic characteristics of the isolates as part of routine screening (e.g., assessing lactose and sucrose fermentation, lysine decarboxylation, hydrogen sulfide production, indole production, and motility, among other properties) (Humphries and Linscott, 2015; International_Organization_for_Standardization, 2017; Feng et al., 2018; Da Silva et al., 2019) without genotyping for the presence of *eae* in *E. albertii* for additional species-specific PCR analysis to fully identify the pathogen (Hyma et al., 2005; Oaks et al., 2010).

We suspect that the existing *E. albertii* biogroups may be outdated and should, therefore, be modified. Some strains initially identified as *Shigella boydii* type 13 because they differ in their key biochemical characteristics (indole production and lysine decarboxylation activity) from the original *E. albertii* strains have been reclassified as *E. albertii* lineages (Hyma et al., 2005). Subsequently, two *E. albertii* biogroups were documented by Nataro et al. (2007). *E. albertii* biogroup 1 lacks indole production, is positive for lysine decarboxylase activity, and is positive for acid production from D-sorbitol (hereafter referred to as indole-negative/lysine-positive/D-sorbitol-positive), while biogroup 2 shows an indole-positive/lysine-negative/D-sorbitol-negative profile. However, several strains have been reported to have characteristic patterns (indole-positive/lysine-positive) that differ from those of biogroups 1 and 2 (Stock et al., 2005). Ooka et al. (2012) found that 96.2% of the *E. albertii* isolates (25/26) tested in their study showed the indole-positive/lysine-positive phenotype, and therefore did not fit into either of the biogroups defined by Nataro et al. (2007). Hence, biogrouping is a helpful tool for identifying non-straightforward bacterial species.

The above-mentioned issues make it necessary to reorganize the biogroups of *E. albertii*. Therefore, the aim of the current study was to identify common characteristics of non-biogroup 1 or 2 strains of *E. albertii*, and all other *E. albertii* strains, in an attempt to aid the detection and identification of this pathogen in clinical settings.

## Results

### Bird Isolates

**FIGURE 1:**
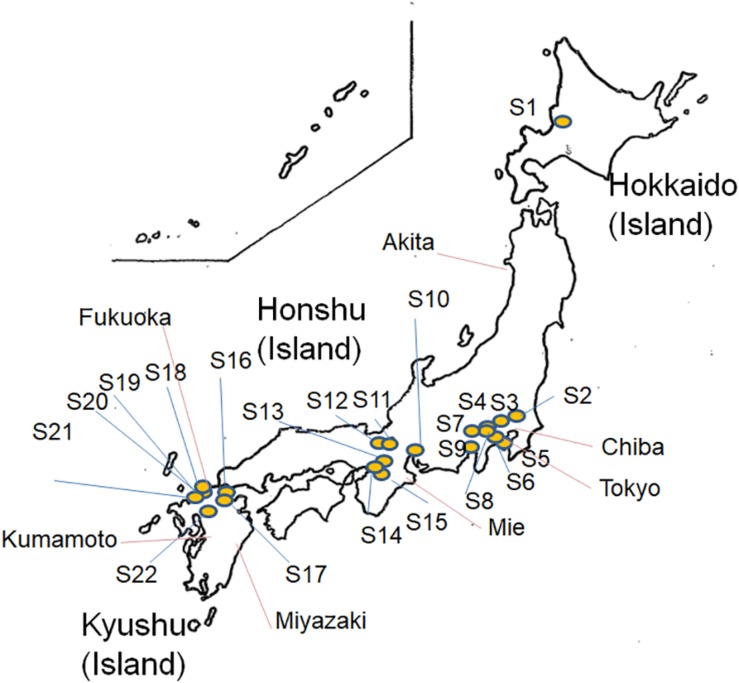
Map of *Escherichia albertii* isolation sites (S1–S22, pigeon isolates and isolates from other sources) in Japan.

As shown in Figure 1, a total of 283 *E. albertii* isolates were obtained from pigeon feces from 22 of 42 sampling sites in Japan (Supplementary Table S1 and Figure 1), while 72 isolates were obtained from other wild birds in Kyushu, Japan (Supplementary Table S1 and Figure 1). All 355 isolates were identified as *E. albertii* using a PCR-based assay (Hyma et al., 2005; Oaks et al., 2010) along with 12 other isolates from wild birds in Hokkaido, totaling 367 bird isolates.

### Pulsed-Field Gel Electrophoresis

**FIGURE 2:**
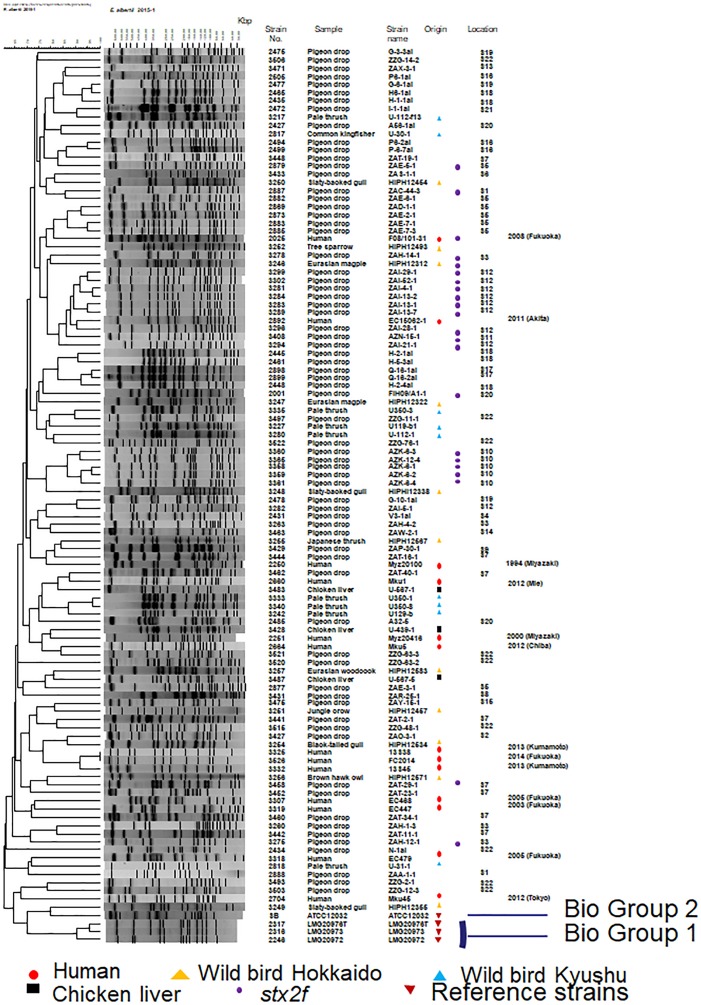
Dendrogram showing the pulsed-field profiles of the *Escherichia albertii* isolates from this study after *Xba*I digestion. Numbers indicate fragment sizes. The origin of each isolate is indicated. The scale bar shows the percentage similarity, as determined by the Dice coefficient analysis. Strains belonging to biogroups 1 and 2 are indicated and the remaining non-indicated strains belong to biogroup 3. A strain (strain 3406; AZN-8) isolated from the pigeon droppings collected at S11 was untypeable so there is no image associated with it in this figure.

A dendrogram based on 107 representative strains (13 from humans, 71 from pigeon feces, 20 from other wild birds, and 3 from retail chicken livers) and four reference strains [*E. albertii* LMG 20976^*T*^, LMG 20972, and LMG 20973, and *S. boydii* ATCC 12032 (reclassified as *E. albertii*) (Hyma et al., 2005)] was constructed. We found that strains from biogroups 1 or 2 belong to a relatively discreet cluster of biogroup 3 strains (Figure 2). No clustering of the human pulsed-field gel electrophoresis (PFGE) profiles is apparent in the dendrogram, a situation that also applies to the wild birds of Hokkaido (Figure 2). Two couples of PFGE profile combinations (2251 and 3428, and 2660 and 3483) from the human strains and the chicken liver strains were found to be located near to each other in the dendrogram.

### Biochemical and Molecular Characterization

**FIGURE 3:**
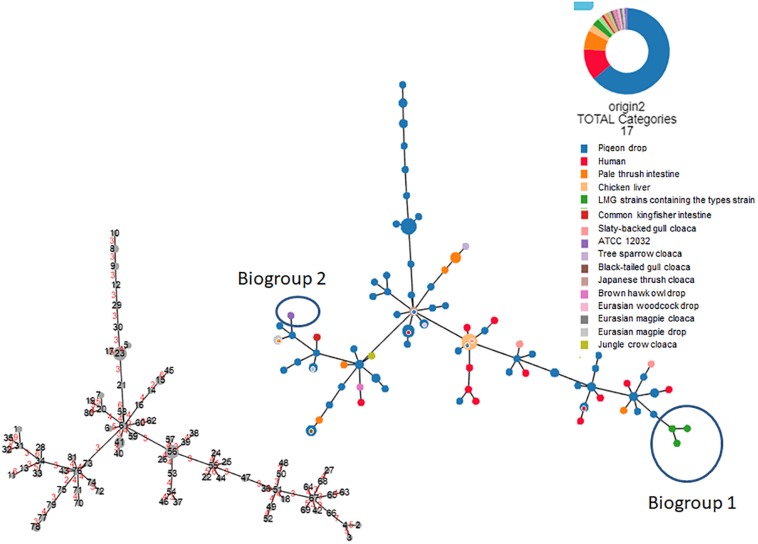
Hierarchical clustering tree (Globally Closest Pir clustering) of 111 *Escherichia albertii* isolates showing 81 profiles based on 77 biochemical characteristics (scaling factor: 7; tree cut-off: 10; n locus variant: 0). The origin of each isolate is indicated. The number of divergent biochemical characteristics between the different profiles is indicated in red on the smaller tree.

Based on the biochemical and molecular testing results, the 107 field strains and four reference strains comprised 81 different profiles (Supplementary Tables S2, S3, Figure 3, and Supplementary Figure S1). Among the non-reference strains, all 107 produced positive results in 18 tests and negative results in 36 tests, with a mixture of positive and negative results for the remaining 26 tests (Supplementary Table S2). All 107 strains were positive for both indole and lysine production. As shown in Figure 3 (based on the 81 profiles and not on the gene information), Supplementary Table S3, and Supplementary Figure S1, none of the 107 field strains could be assigned to either of the biogroups (biogroups 1 and 2) defined by Nataro et al. (2007) (biogroup 1: indole-negative/lysine-positive/D-sorbitol-positive; biogroup 2: indole-positive/lysine-negative/D-sorbitol-negative).

We were able to use lysine decarboxylase activity and indole production characteristics to divide all 111 strains into three biogroups: 1, 2, and non-1 or 2. All 111 strains were negative for 35 tests and positive for 16 tests (Supplementary Table S2). Interestingly, only motility, esterase (C4) activity, and β-glucuronidase activity could be used to successfully discriminate the *E. albertii* strains from the *E. coli* ATCC11775^*T*^ reference strain. As shown in Figure 3 and Supplementary Figure S1, the 111 strains showed 81 different profiles based on the 77 characteristics we assessed. Profiles 23 (*n* = 6), 56 (*n* = 6), 41 (*n* = 4), 15 (*n* = 3), 78 (*n* = 3), 8 (*n* = 2), 9 (*n* = 2), 32 (*n* = 2), 33 (*n* = 2), 47 (*n* = 2), 49 (*n* = 2), 51 (*n* = 2), 59 (*n* = 2), 61 (*n* = 2), 65 (*n* = 2), 67 (*n* = 2), 74 (*n* = 2) and 76 (*n* = 2) included multiple strains (Supplementary Figure S1). Profile 56 was the dominant profile associated with strains from several different sources (pigeon droppings, slaty-backed gull droppings, and retail chicken livers). Molecular testing revealed that 19.8% (22/111), 100%, and 100% of the tested strains were positive for *stx2f*, *eae*, and *cdt* genes, but no *stx* genes other than *stx2f* were detectable in the strains (Supplementary Table S2).

### Multi-Locus Sequence Analysis

**FIGURE 4:**
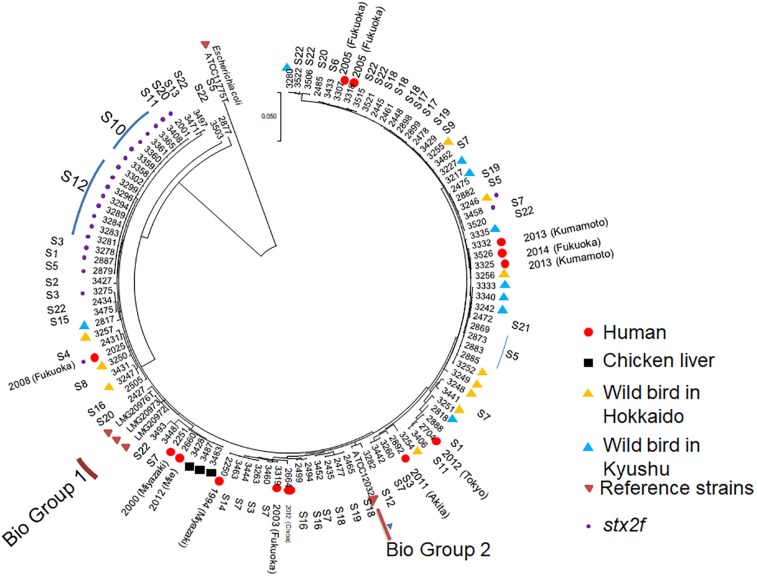
Multi-locus sequence analysis-based phylogenetic tree for 111 *Escherichia albertii* isolates and ATCC11775^*T*^, a representative *Escherichia coli* strain, using the maximum-likelihood method. The evolutionary history was inferred using the maximum-likelihood method based on the general time-reversible model. Initial tree(s) for the heuristic search were obtained automatically by applying the Neighbor-Join and BioNJ algorithms to a matrix of pairwise distances estimated using the maximum composite likelihood approach, and then selecting the topology with the superior log likelihood value. A discrete gamma distribution was used to model evolutionary rate differences among sites [five categories (+G, parameter = 0.1325)]. The rate variation model allowed for some sites to be evolutionarily invariable [(+I), 45.6617% sites]. The tree is drawn to scale, with branch lengths measured by the number of substitutions per site. The analysis involved 112 nucleotide sequences. Included codon positions were 1st+2nd+3rd+Non-coding. Isolates belonging to biogroups 1 and 2 are indicated, while the remaining non-indicated isolates belong to biogroup 3.

As shown in Figure 4, multi-locus sequence analysis (MLSA) based on seven loci showed that multiple clones were present among the 107 strains tested in the present study. A total of 18 strains harboring the Shiga toxin 2f gene (*stx2f*) showed no variation across the entire 3,423-bp concatenated sequence despite being obtained from seven different locations, while pigeon strains 2877 and 3503 showed relatively unique sequences. However, there was not a huge degree of separation between biogroup 1 and 2 strains and non-biogroup 1 and 2 strains based on the MLSA results (Figure 4). Two human strains (strain numbers 2251 and 2660) and three retail chicken liver strains (strain numbers 3428, 3487, and 3483) also had identical sequences across the 3,423-bp concatenated fragment (Supplementary Table S4). The strains from the wild birds in Hokkaido clustered into in several clades.

## Discussion

The results of the present study suggest that isolates of the emerging zoonotic pathogen *E. albertii*, which belong to non-biogroup 1 or 2, should be assigned to biogroup 3. Importantly, none of the strains collected from Japan in the present study showed characteristics consistent with those of biogroups 1 or 2. Moreover, isolates from the United States (Oaks et al., 2010; Nimri, 2013), Australia (Oaks et al., 2010), Scotland (Oaks et al., 2010), and Canada (Oaks et al., 2010; Maheux et al., 2014) have also been shown to have the indole-positive/lysine-positive phenotypic characteristics of the non-biogroup 1 and 2 strains examined in our study. Therefore, *E. albertii* non-biogroup 1 and 2 strains appear to be fairly common worldwide. We found that biogroup 1, biogroup 2, and non-biogroup 1 and 2 strains were only differentiated by indole production and lysine decarboxylase activity. In line with this finding, indole production and lysine decarboxylase activity were found to be key characteristics for identifying enterobacterial strains in clinical laboratories (Barrow and Feltham, 2004; International_Organization_for_Standardization, 2017; Feng et al., 2018). Generally, differences in these two characteristics between isolates usually mean that they are different species. Therefore, variations in these characteristic patterns among *E. albertii* biogroups could cause difficulties with pathogen identification. Biogrouping is particularly important for pathogens because classification is an important step for accurate identification (Barrow and Feltham, 2004). As such, we propose that non-biogroup 1 and 2 *E. albertii* strains should be assigned to biogroup 3.

*Enterobacteriaceae* isolates showing certain characteristics should be examined for potential identification as *E. albertii*. In the present study, negative motility, positive esterase (C4) activity, and negative β-glucuronidase activity were discriminative characteristics for distinguishing *E. albertii* from *E. coli* ATCC11775^*T*^. However, esterase (C4) is not an easy test to conduct as part of a standard examination protocol. Therefore, based on our findings (Supplementary Table S2), we recommend that isolates with the phenotypic properties of being mobility-negative (sulfide-indole-motility medium, 37°C), hydrogen sulfide production-negative (triple sugar iron medium), acid production-negative (from xylose), and negative for β-glucuronidase activity, should be examined further using an *E. albertii*-specific PCR assay (Hyma et al., 2005; Oaks et al., 2010). In addition, the designation of group criteria for biogroups 1, 2, and non-1 or -2, as indole-negative/lysine-positive, indole-positive/lysine-negative, and indole-positive/lysine-positive, respectively, will further contribute to the identification of isolates as *E. albertii*. Isolation methods for *E. albertii* should also be improved in the interests of public health. The biochemical/molecular characterization and MLSA results from the current study reveal the presence of close relationships between some human strains and retail chicken liver strains, suggesting that chicken livers might be a vehicle for transmission of the pathogen. This observation is strengthened by the results of a study on *Campylobacter jejuni* (Duarte et al., 2019). To date, *E. albertii* has been isolated from pigs, cats, seals, and various birds, indicating that these animals might be reservoirs for the pathogen (Oaks et al., 2010; Hinenoya et al., 2014; Grillová et al., 2018). In addition, the isolation of strains from pork, mutton, duck meat, and chicken meat suggests that consumption of meat from infected food-producing animals is a likely route for *E. albertii* transmission (Maeda et al., 2015; Wang et al., 2016). However, gaining a comprehensive understanding of potential reservoirs and vehicles of transmission is hindered by inadequate *E. albertii* isolation methods (Maeda et al., 2014). Therefore, more effort should be put into developing effective isolation methods for *E. albertii*.

Additional molecular genetics-based approaches are needed to discern the phylogenetic differences occurring between the different biogroups. The molecular analysis methods (MLSA and PFGE) used in this study generated data that poorly reflected the relationship between genetic typing and biogrouping in the *E. albertii* strains we tested. Lysine decarboxylase activity profiling was a key feature of the biogrouping analyses in the present study. Loss of this enzyme activity resulted in the enteropathogen *Shigella* evolving as a distinct entity from non-pathogenic *E. coli* (which is lysine decarboxylase activity positive) because cadaverine, a product of decarboxylated lysine, inhibits enterotoxin activity in *Shigella* (Janda and Abbott, 2006). Lysine decarboxylase activity negativity or positivity in *E. albertii* might be related to the pathogenic evolution of the two bacterial genera. Therefore, molecular phylogenetic approaches based on whole genome sequencing (Ooka et al., 2015) might better elucidate the relationships between the phylogenetic groups and the biogroups in the zoonotic pathogen *E. albertii*.

In conclusion, this study provides evidence suggesting that non-biogroup 1 and 2 strains of *E. albertii*, an emerging zoonotic pathogen, should be assigned to biogroup 3. While a poor relationship was seen between the genetic typing and biogrouping results for *E. albertii* using MLSA and PFGE, biogroup classification is clearly an important step in the identification process. In addition, our grouping criteria for biogroup 1 (indole-negative/lysine-positive), biogroup 2 (indole-positive/lysine-negative), and biogroup 3 (indole-positive/lysine-positive) strains is an enhancement to previous work (Nataro et al., 2007), and makes a positive contribution to the identification of isolates as *E. albertii*. We recommend that isolates displaying mobility negativity (sulfide-indole-motility medium, 37°C), hydrogen sulfide production negativity (triple sugar iron medium), acid production negativity from xylose, β-glucuronidase activity negativity (4-methylumbelliferyl-beta-D-glucuronide), and with indole production and lysine decarboxylase activity profiles consistent with one of the three biogroups, should be examined further using an *E. albertii*-specific PCR assay.

## Materials and Methods

### Pigeon Isolates From Japan and Wild Bird Isolates (Other Than Pigeons) From Kyushu, Japan

Pigeon droppings were collected as described previously (Murakami et al., 2014a) (Supplementary Table S1), whereas the intestines from wild birds (Supplementary Table S1) were minced using sterilized scissors. Pigeon droppings were suspended in 10 ml of buffered peptone water (BPW; Oxoid Ltd., Basingstoke, United Kingdom), and the minced intestine samples were homogenized with nine volumes of BPW using a Stomacher paddle blender (Seward, Worthing, United Kingdom). All samples were then cultured at 42°C for 16 h. Supernatants from each of the cultures were assayed using a PCR-based method with *lysP* primers as previously described (Maeda et al., 2015). Aliquots (1 ml) from each of the *lysP*-positive samples were then cultured in 10-ml volumes of modified BPW supplemented with 2.3 ml of XLT-4 agar supplement (Becton, Dickinson and Company, Franklin Lakes, NJ, United States) per liter of BPW at 42°C for 18 h. Dilutions of the resulting cultures were then inoculated onto DHL agar (Nissui Pharmaceutical Co., Tokyo, Japan) and MacConkey agar (Becton, Dickinson and Company) plates supplemented with L-xylose and L-rhamnose (both 1% v/w) (Wako Pure Chemical Industries, Osaka, Japan). Putative *E. albertii* isolates, which formed glistening, white colonies, were isolated and then identified using PCR-based methods, as previously described (Murakami et al., 2014b).

### Other Isolates

As shown in Supplementary Table S1, *E. albertii* isolates from outbreak-related patients (*n* = 32), asymptomatic cases (*n* = 5), wild birds in Hokkaido (*n* = 12), and retail chicken livers (*n* = 10) were also examined in this study. *E. albertii* strains LMG 20976^*T*^, LMG 20972, and LMG 20973 (all biogroup 1), *S. boydii* strain ATCC 12032 (reclassified as *E. albertii* biogroup 2) (Hyma et al., 2005), and *E. coli* strain ATCC 11775^*T*^ were included as reference strains.

### PFGE and Representative Isolate Selection

A total of 414 isolates were digested with *Xba*I (Takara Bio Inc., Otsu, Japan) for representative strain selection, as described previously (Murakami et al., 2017). Similarity and cluster analyses were performed using the Dice coefficients of similarity analysis and an unweighted pair group method with average linkage in FPQuest software (Bio-Rad Laboratories, Hercules, CA, United States). Reference strains (*n* = 4) were also examined with PEGE. Of the 414 field isolates (37 human, 367 bird, and 10 chicken liver isolates, Supplementary Table S1), 105 were selected as representative isolates based on their unique PFGE profiles. In addition, one of the two pigeon isolates that was found to be non-typeable by PFGE was also selected as a representative strain (No. 3406 in Figure 2). Isolate number 3526 (Figure 2), which was collected from a patient co-infected with human norovirus, showed the same PFGE profile as another outbreak isolate (No. 3325, Figure 2) and was therefore selected for further investigation because of the difference in isolation year. The 107 isolates (13 from humans, 71 from pigeon feces, 20 from other wild birds, and 3 from retail chicken livers) obtained from 92 sources (13 human fecal samples, 60 pigeon fecal samples, 19 other wild bird samples, and 2 retail chicken liver samples) were further examined, as were the reference strains comprising *E. albertii* LMG 20976^*T*^, LMG 20972, and LMG 20973 and *S. boydii* ATCC 12032 (re-classified as *E. albertii*) (Hyma et al., 2005).

### Biochemical and Molecular Characterization

Isolates were examined for biochemical and molecular characteristics using culture-based methods, commercial kits, and PCR-based methods for virulence gene detection. The culture-based methods included lysine (24 h), indol (24 h), mobility (40 h), citrate (Simmons) (4 days), arginine (4 days), ornithine (4 days), Voges-Proskauer (24 h), and acetate (2 days) assays conducted at 37°C, as previously described (Barrow and Feltham, 2003). All examinations were carried out triplicate. Commercial Api 50CHE (BioMérieux, Lyon, France) and Api Zym (BioMérieux) testing kits were used according to the manufacturer’s instructions. All testing with commercial kits was performed in duplicate.

PCR-based methods were used to determine the presence of virulence genes in each of the strains. Specific primers were used to screen for *eae* and *aggR*, as described previously (Kobayashi et al., 2002). *cdt* was PCR-amplified using previously described methodology (Pickett et al., 1996). Two pairs of primers targeting *stx2f*, the sequences of which were described previously by Nakao et al. (2002) and Schmidt et al. (2000) were used for double checking. Strains designated *stx2f*-positive produced positive results using both sets of primers. Commercial primers (Takara Bio Inc., Otsu, Japan) for Shiga toxin-associated genes (except *stx2f*) were also used. All PCR assays were carried out triplicate.

The 77 biochemical characterization (items as shown in Supplementary Table S2) results were visualized using Phyloviz Online^1^ (Ribeiro-Gonçalves et al., 2016) (Figure 3).

### MLSA

The MLSA data for five strains [reference strains and strain No. 2025 (F08/101-31)] were obtained from our previous study (Murakami et al., 2014b). MLSA was also used for the remaining 106 of the 111 strains by targeting the seven following housekeeping genes: *adk* (adenylate kinase), *fumC* (fumarate hydratase), *gyrB* (DNA gyrase), *icd* (isocitrate/isopropylmalate dehydrogenase), *mdh* (malate dehydrogenase), *purA* (adenylosuccinate dehydrogenase), and *recA* (ATP/GTP binding motif). Sequencing was performed as previously described (Murakami et al., 2014a). The allele sequences from each strain were then concatenated in the order *adk*–*fumC*–*gyrB*–*icd*–*mdh*–*purA*–*recA*, resulting in a final composite length of 3,423 bp. Sequences from the remaining five strains, which were analyzed in a previous study, were also included (Murakami et al., 2014b). The resulting concatenated sequences were aligned using ClustalW (Thompson et al., 1994), and a phylogenetic tree was constructed using the maximum-likelihood method with the GTR+G model of evolution (and 1,000 bootstrap replicates). Evolutionary analyses were conducted in MEGA7 (Kumar et al., 2016). The final sequences were deposited in the DNA Data Bank of Japan under accession numbers LC320162–LC320663 and LC320676–LC320957. As the sequencing was not performed using a next generation sequencer, the strains were not assigned sequence types. However, all the data were examined using the EnteroBase database^2^.

## Data Availability

The datasets for this study can be found in the Supplementary Tables S3, S4.

## Ethics Statement

This study was carried out in strict accordance with the guidelines of the Regulations for the Ethical and Humane Use of Experimental Animals at Fukuoka Institute of Health and Environmental Sciences, which is based on domestic standards, and the study was approved by the Animal Ethics Committee of Fukuoka Institute of Health and Environmental Sciences under permit number 250802.

## Author Contributions

KM conducted all the experimental work. EM-M, HK, MH, TI, WS, TK, KK, NS, FM, TS, YK, TI, MO, and KO assisted with project development and data analysis. YE assisted with the computer analysis. SF assisted with the manuscript writing.

## Conflict of Interest Statement

The authors declare that the research was conducted in the absence of any commercial or financial relationships that could be construed as a potential conflict of interest.

## Footnotes

1 https://online.phyloviz.net/index

2 https://enterobase.warwick.ac.uk/species/ecoli/allele_st_search
