# Supplementary material for: Non-biogroup 1 or 2 Strains of the Emerging Zoonotic Pathogen Escherichia albertii, Their Proposed Assignment to Biogroup 3, and Their Commonly Detected Characteristics
Source: Front Microbiol. 2019 Jul 5;10:1543. doi: 10.3389/fmicb.2019.01543 (PMC6624678; doi:10.3389/fmicb.2019.01543)
Supplement: Supplementary file 5 [file Data_Sheet_1.PDF]

81 patterns

Scaling factor: 7
Tree cut-off: 10
NLV graph: 0

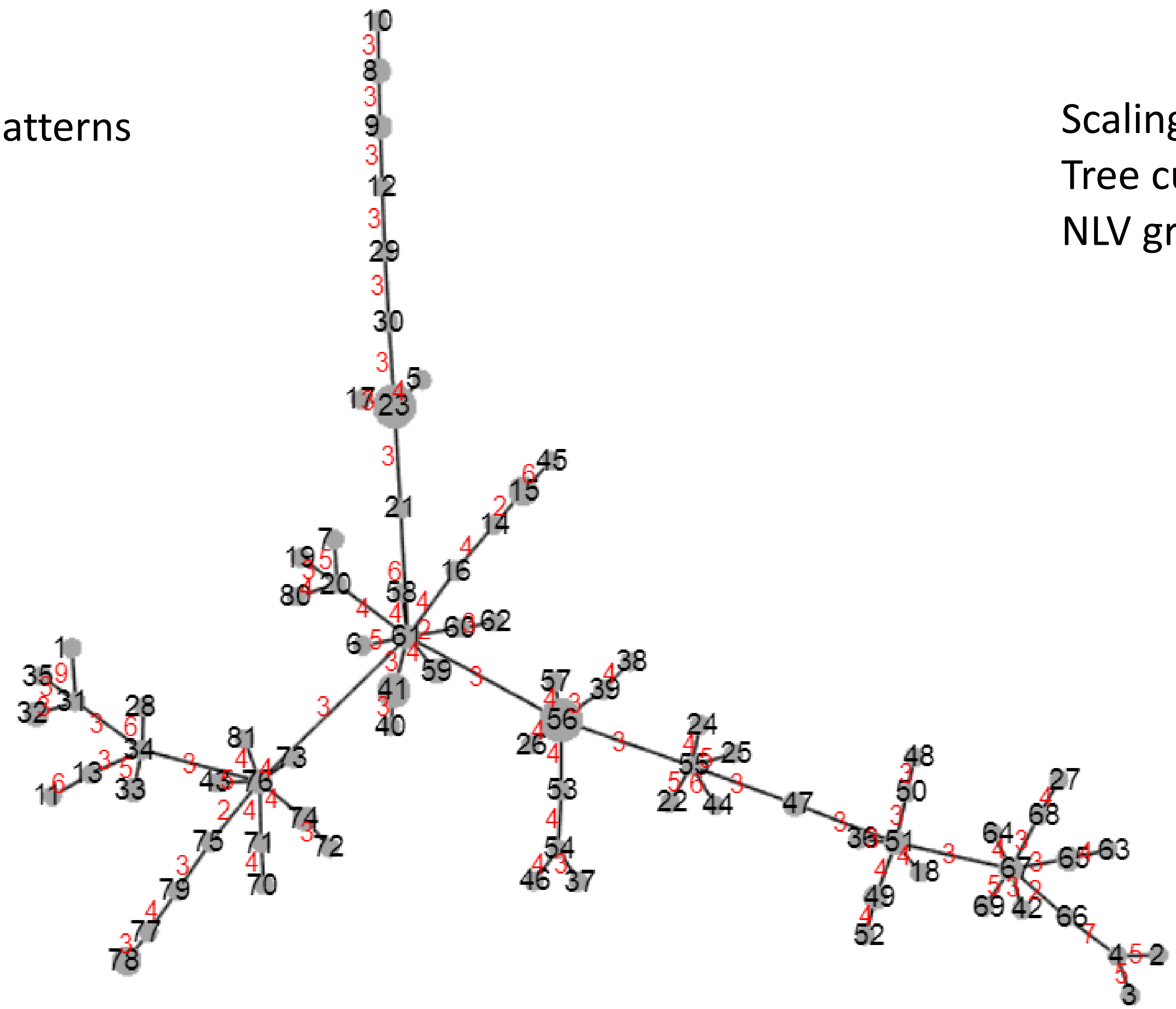

Supplemental Fig. 1. Hierarchical clustering tree (Globally Closest Pir clustering) of a total of 111 *Escherichia albertii* strains showing 81 profiles based on 77 biological characteristics

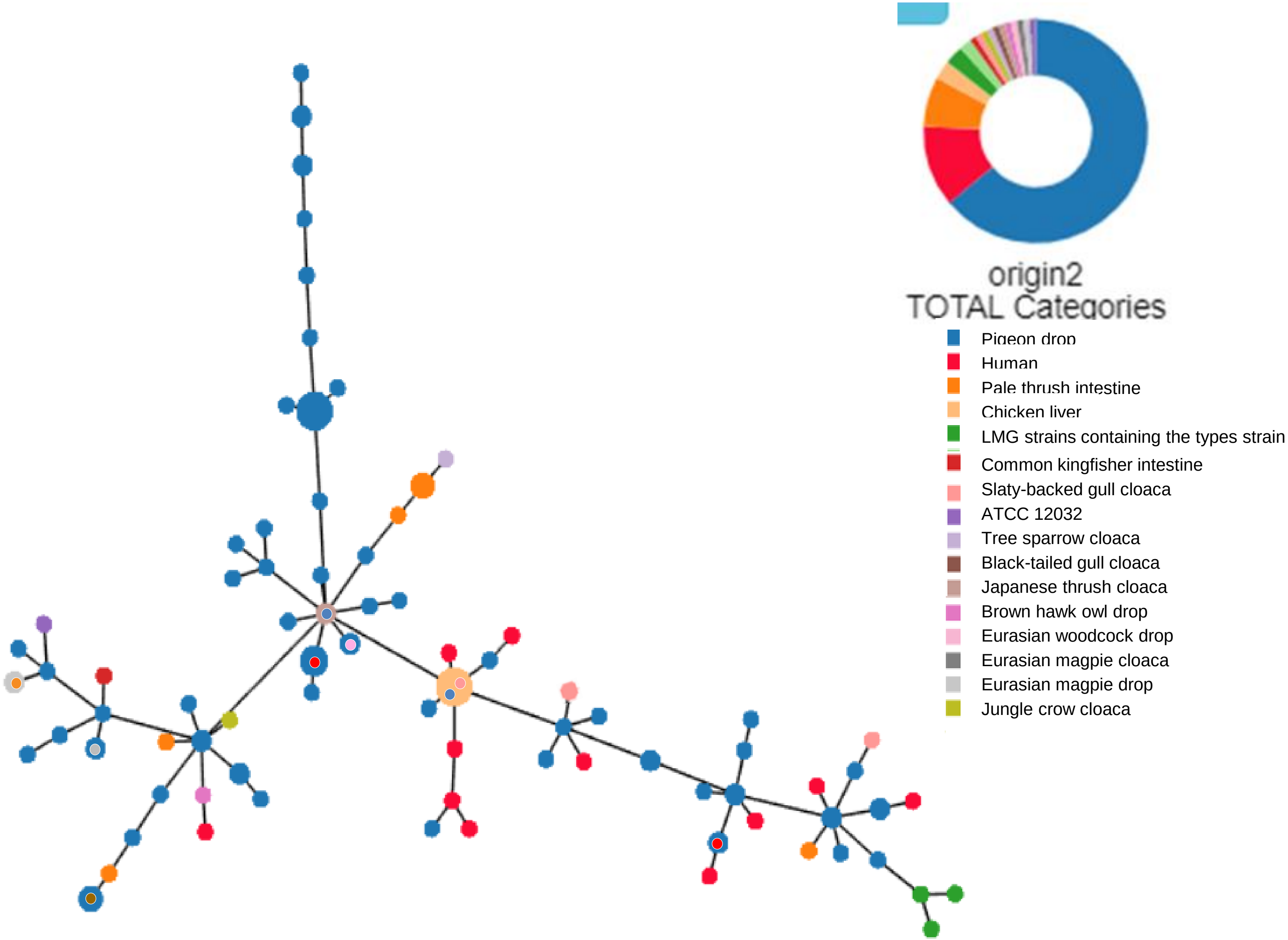

origin2
TOTAL Categories
Pigeon drop
Human
Pale thrush intestine
Chicken liver
LMG strains containing the types strain
Common kingfisher intestine
Slaty-backed gull cloaca
ATCC 12032
Tree sparrow cloaca
Black-tailed gull cloaca
Japanese thrush cloaca
Brown hawk owl drop
Eurasian woodcock drop
Eurasian magpie cloaca
Eurasian magpie drop
Jungle crow cloaca

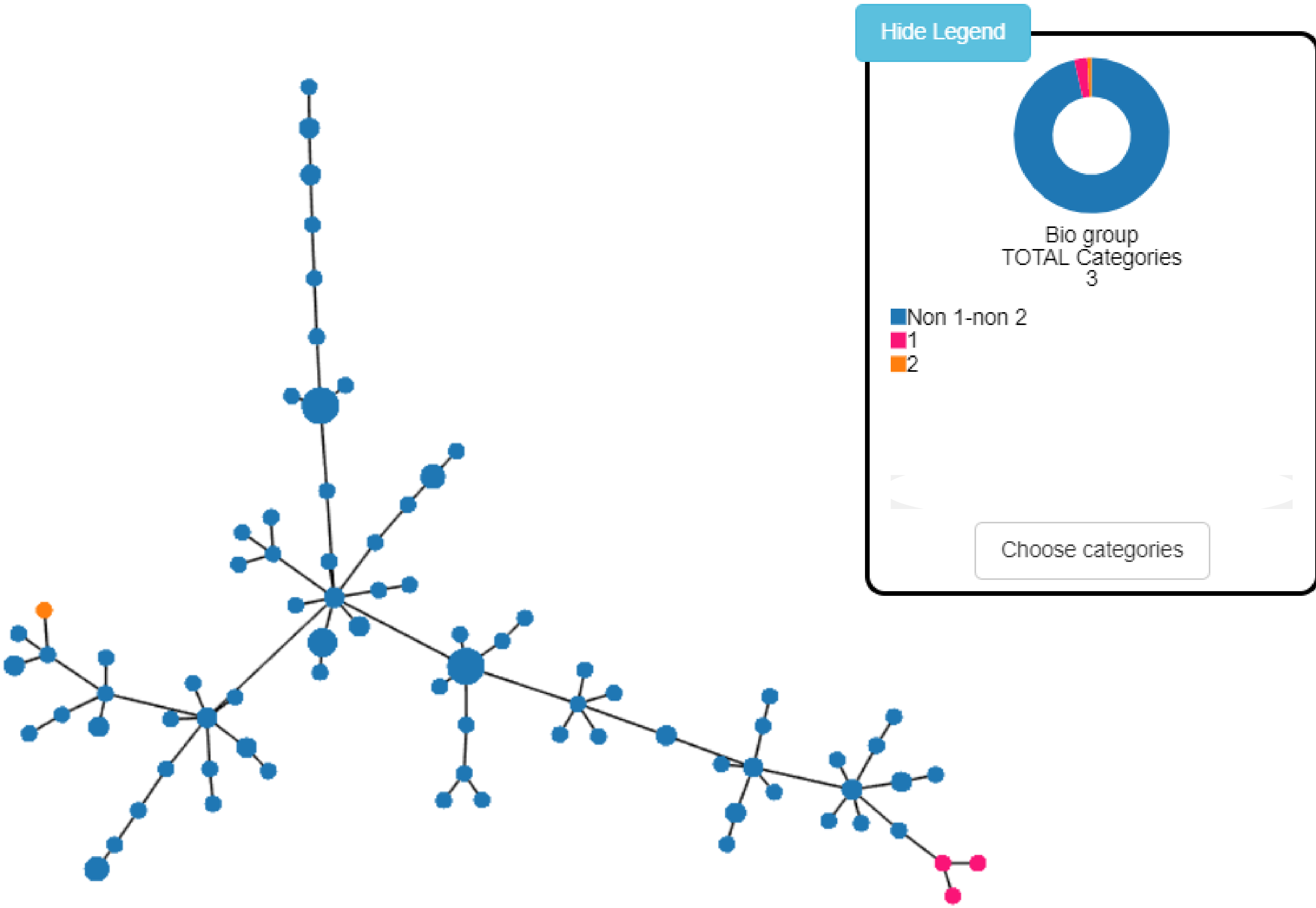
Hide Legend
Bio group
TOTAL Categories
3
Non 1-non 2
1
2
Choose categories

Bio-groups

eae
cdt
L-arabinose
Ribose
D-galactose
D-glucose
D-fructose
D-mannose
D-manitolol
N-acetyle glucosamine
Gluconate
Alkaline phosphatase
Esterase (C4)
Leucine allyamidase
Acid phosphatese
Beta-galactosidase

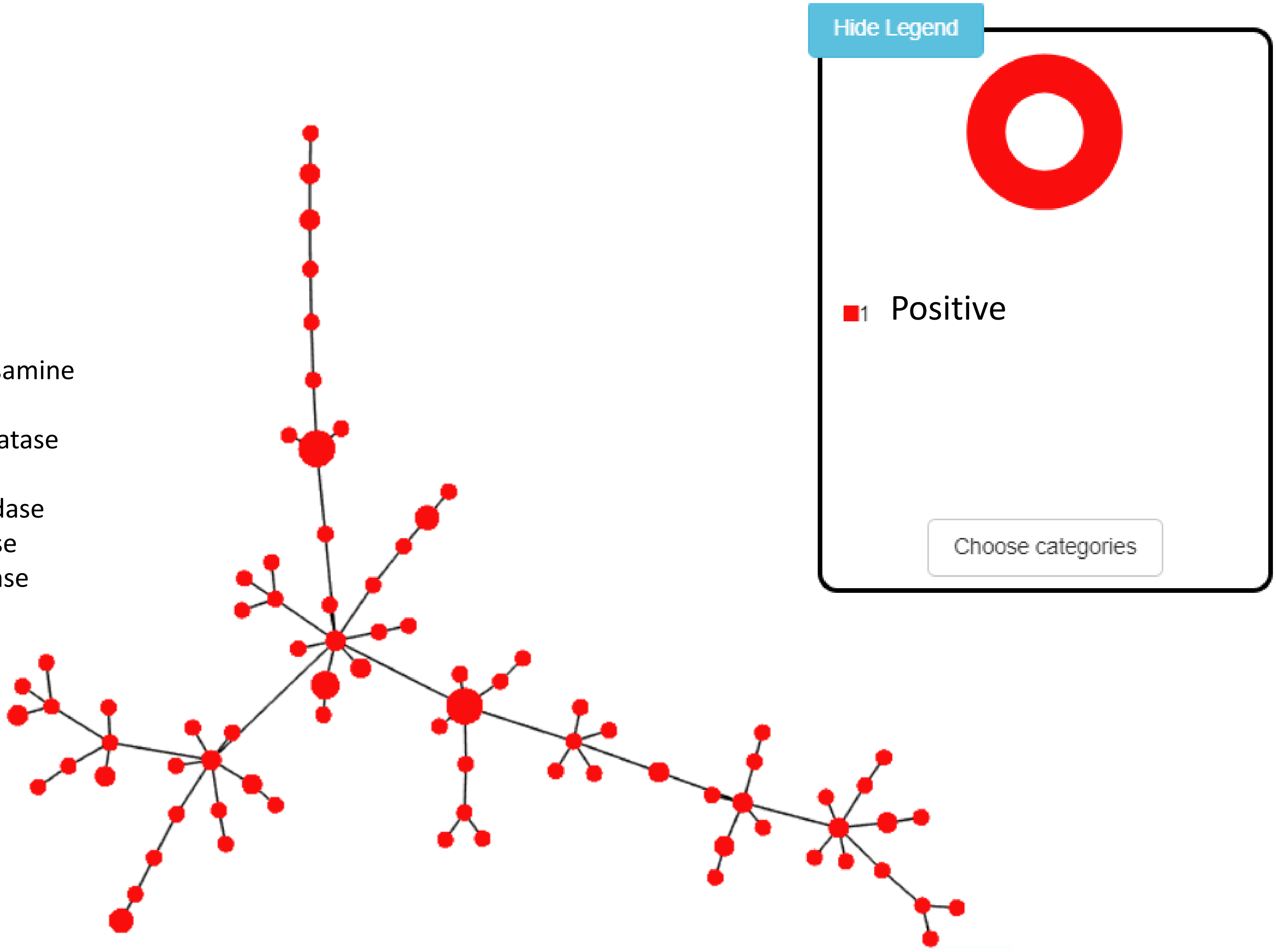

All strain showed positive results in these characteristics.

H_2S
Mobillty
Citrate
VP
Erythritol
D-xylose
L-xylose
D adonitol
Methyle-beta-d-xylopyranose
L-rhamnose
Dulcitol
Inositol
Metyl alpa D Mannopyranosude
Amygdalin
Arbutin
Esculin femic citrate
Lactose
Inullin
D-melezitose
Starch
Glycogen
D-turanose
D-xylose
D-fucose
D-arabitol
L-arabitol
Esterase lipase (C8)
Lipase (C4)
Chymotrypsin
Alpha galactodidase
Beta-glucronidase
Beta-glucosidase
N-acetyle-beta-glucosaminidase
Alpha-mannosidase
Alpha-fucosidase

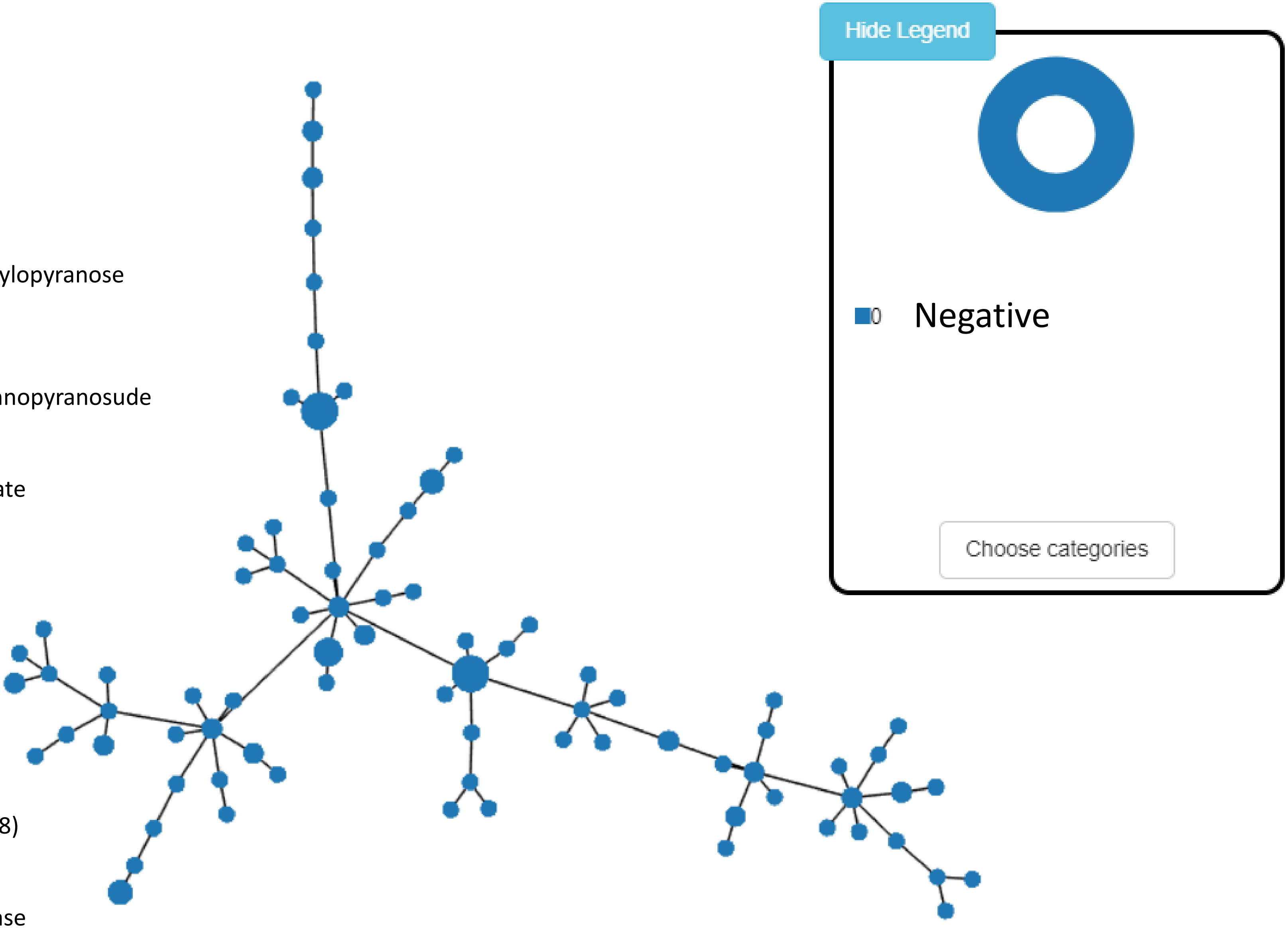

All strain showed Negative results in these characteristics.

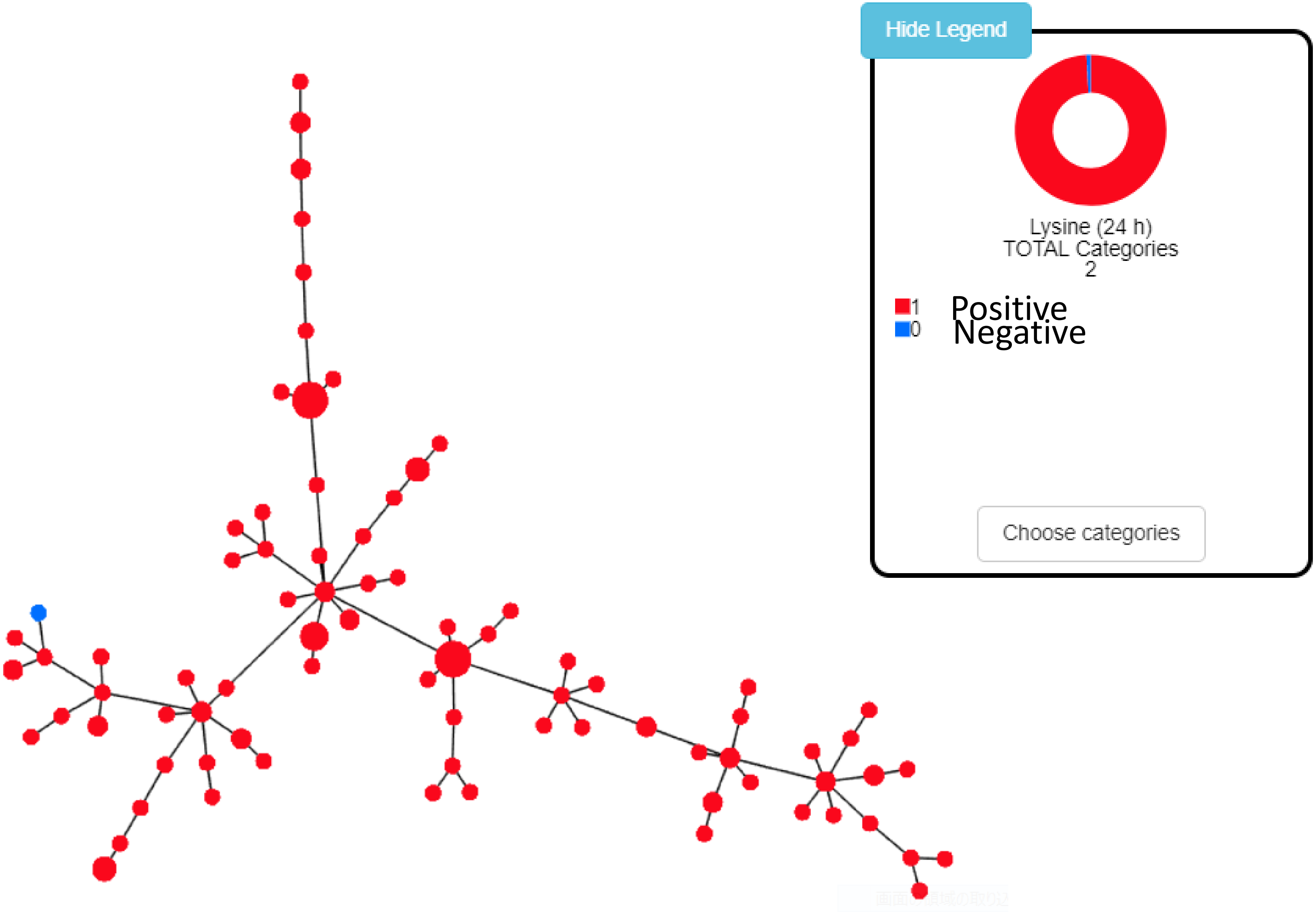

Lysine (24 h) #2 in Table 2

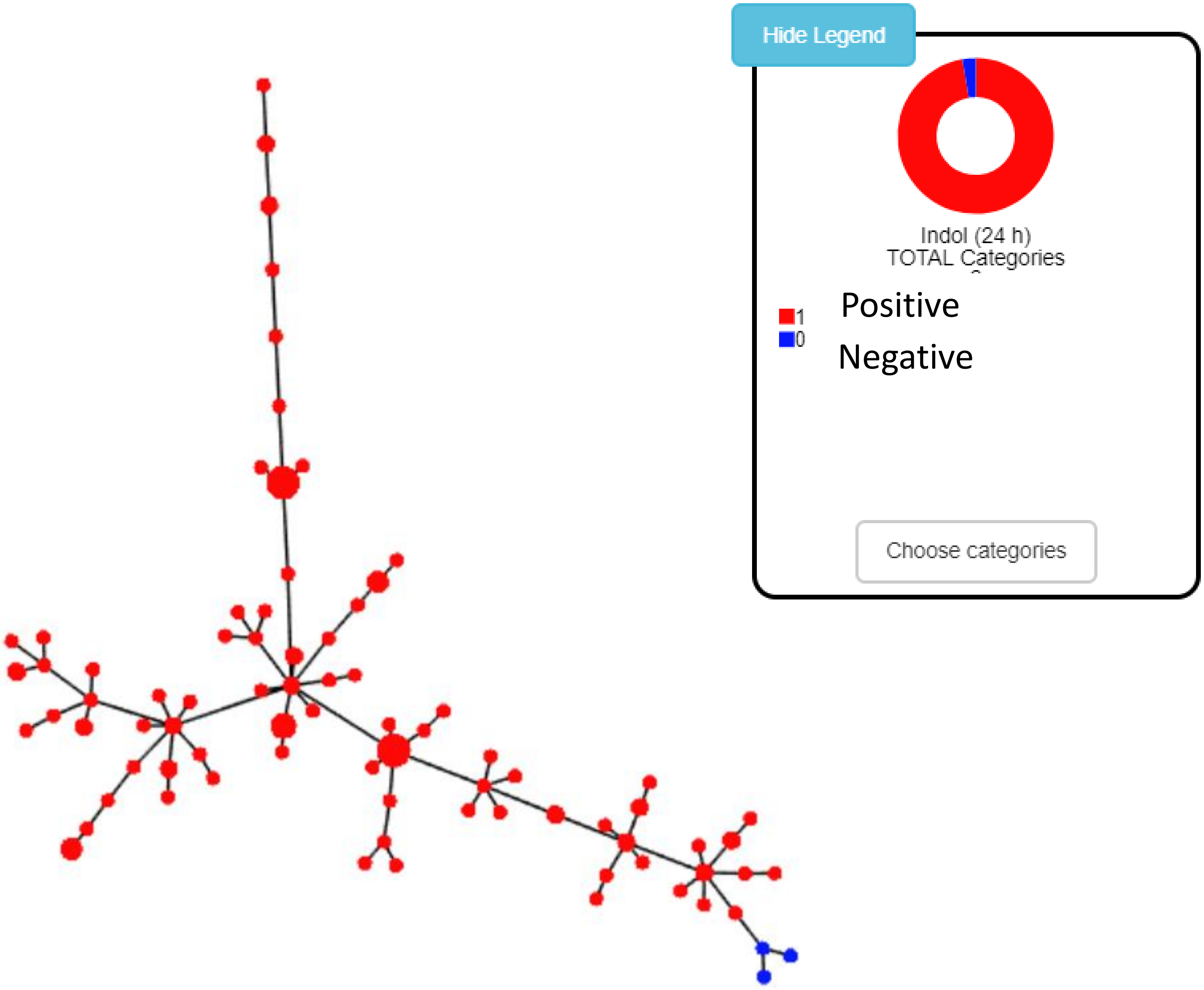

Indole (24 h) #3 in Table 2

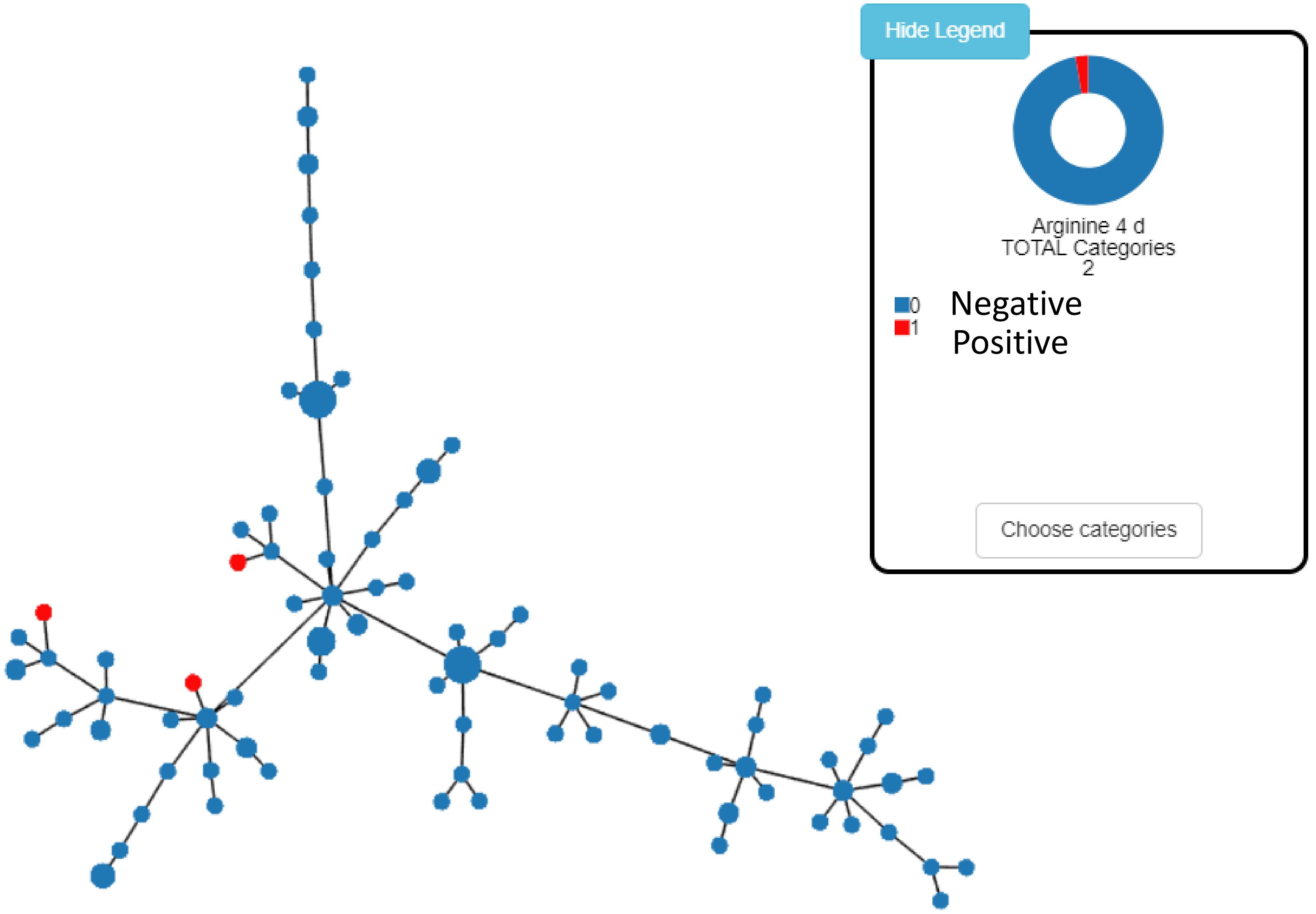

Arginine (4 days)

#6 in Table 2

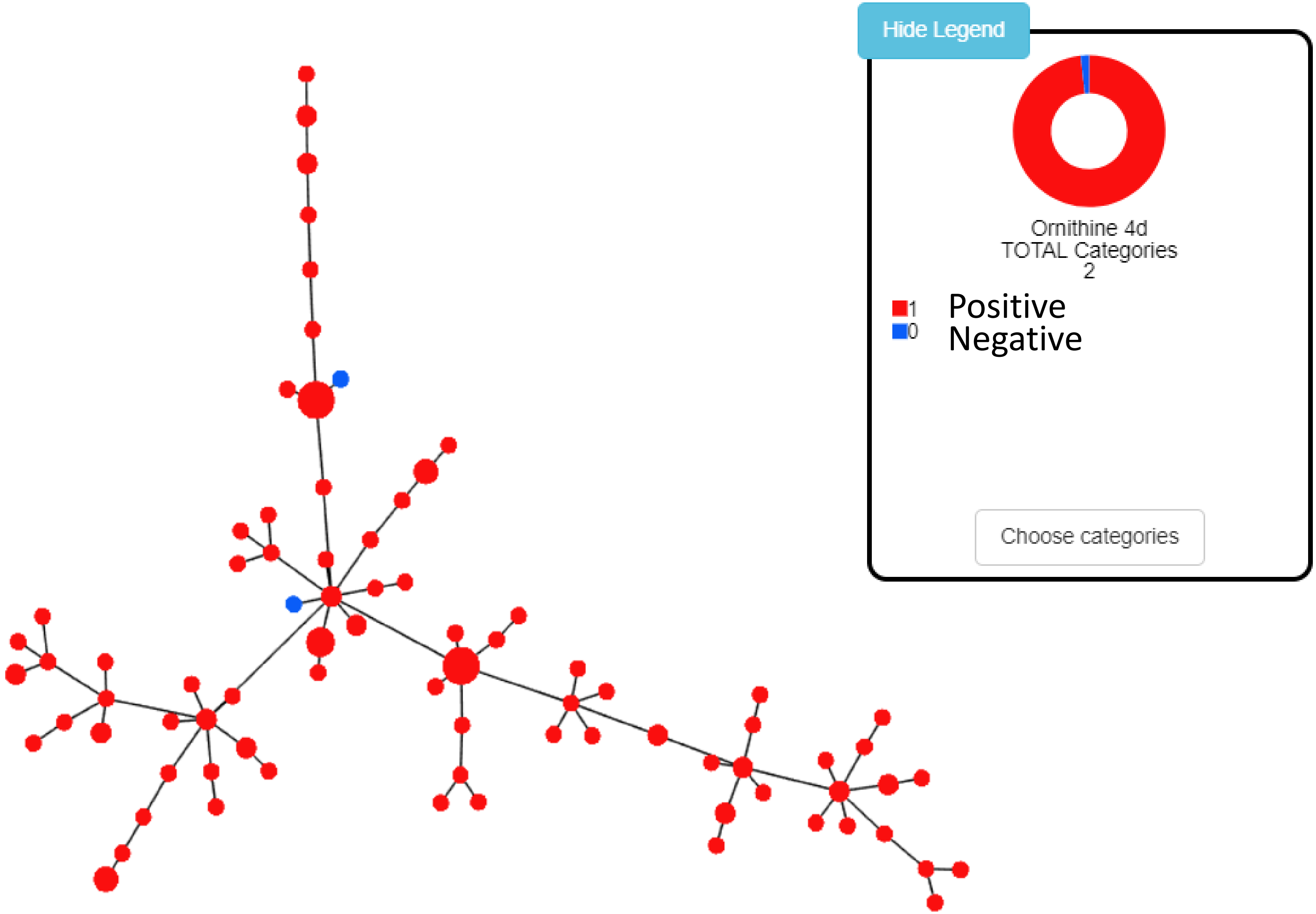

Ornithine (4 days) #7 in Table 2

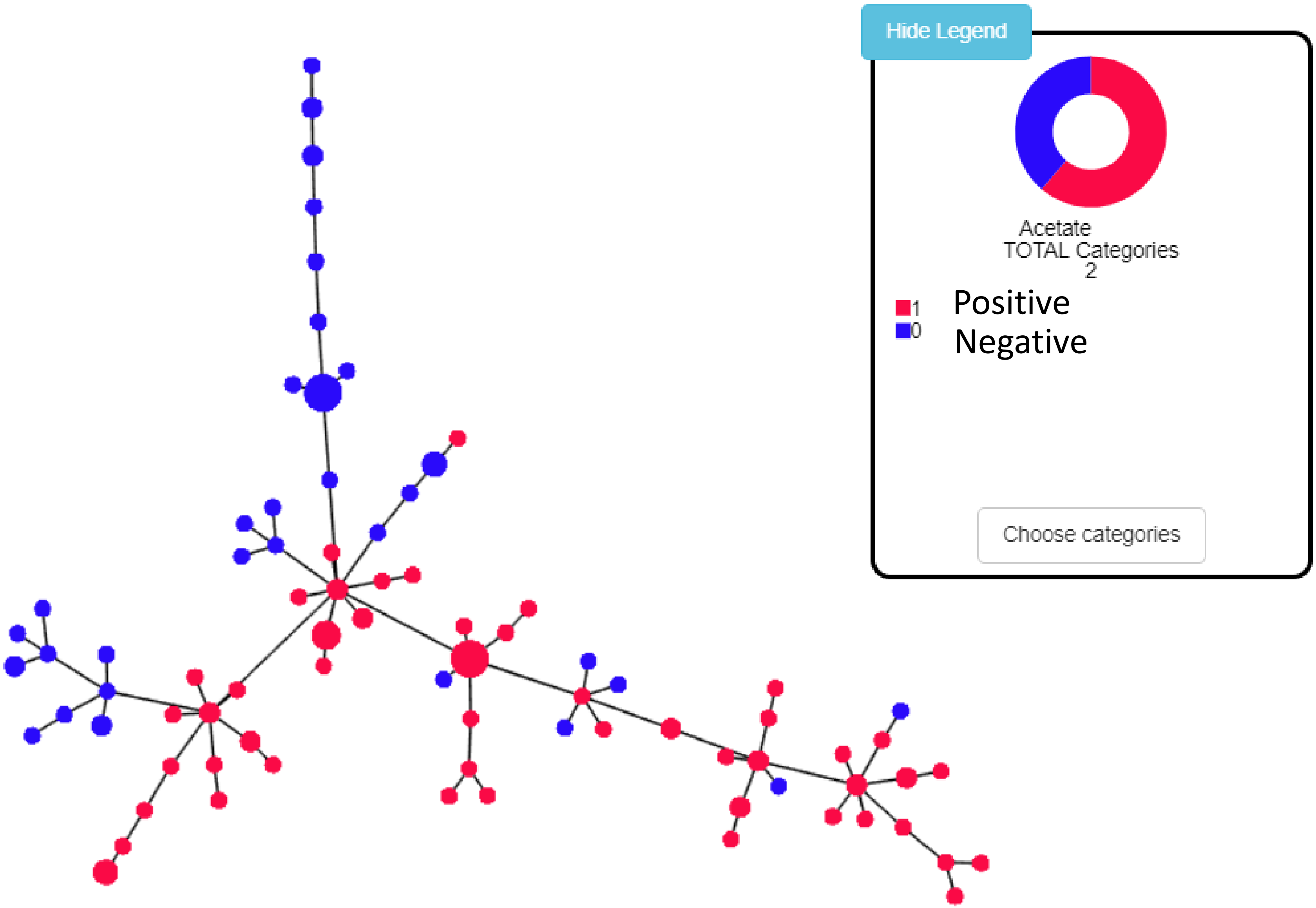

Acetate (2 days) #9 in Table 2

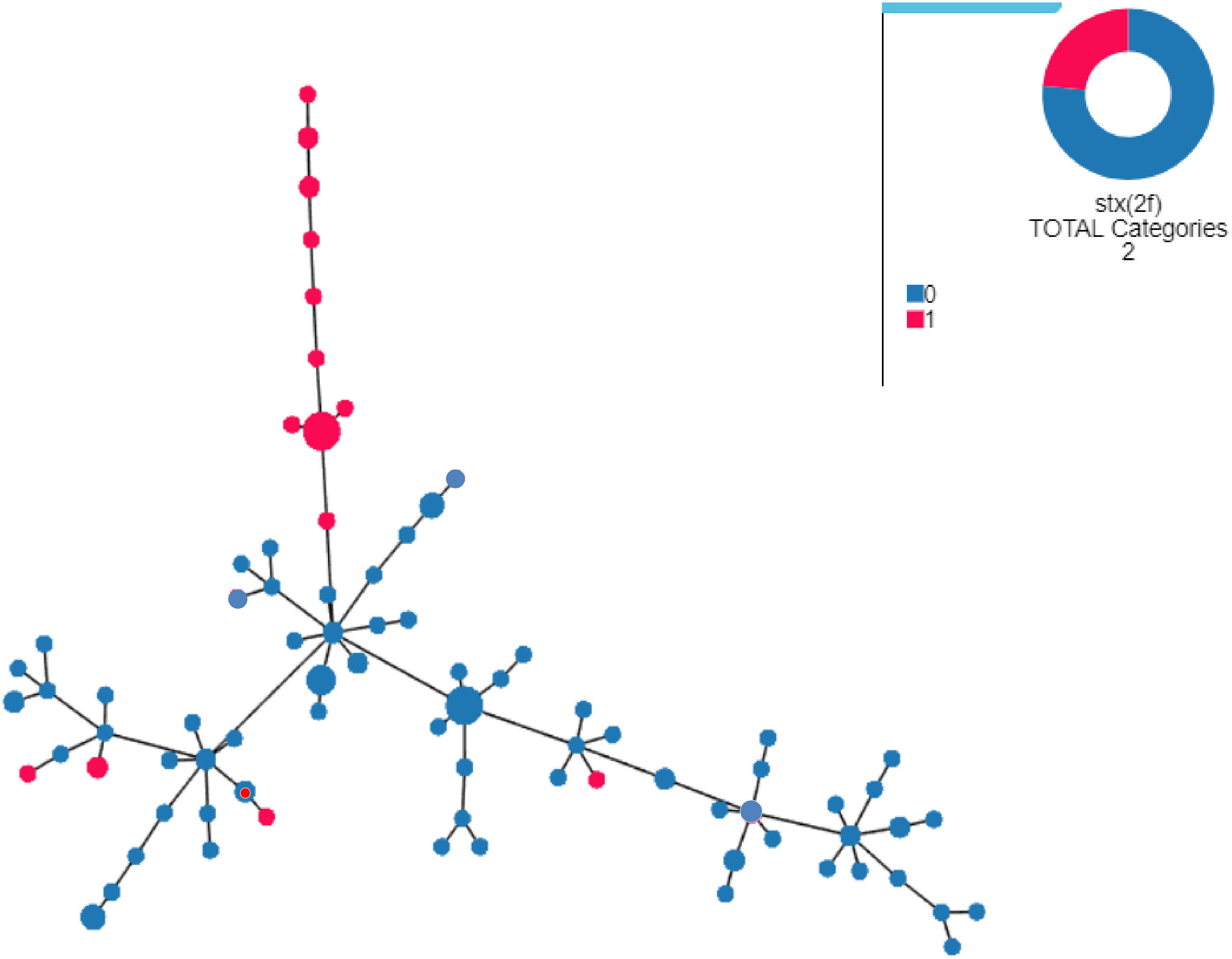

stx2f #10 in Table 2

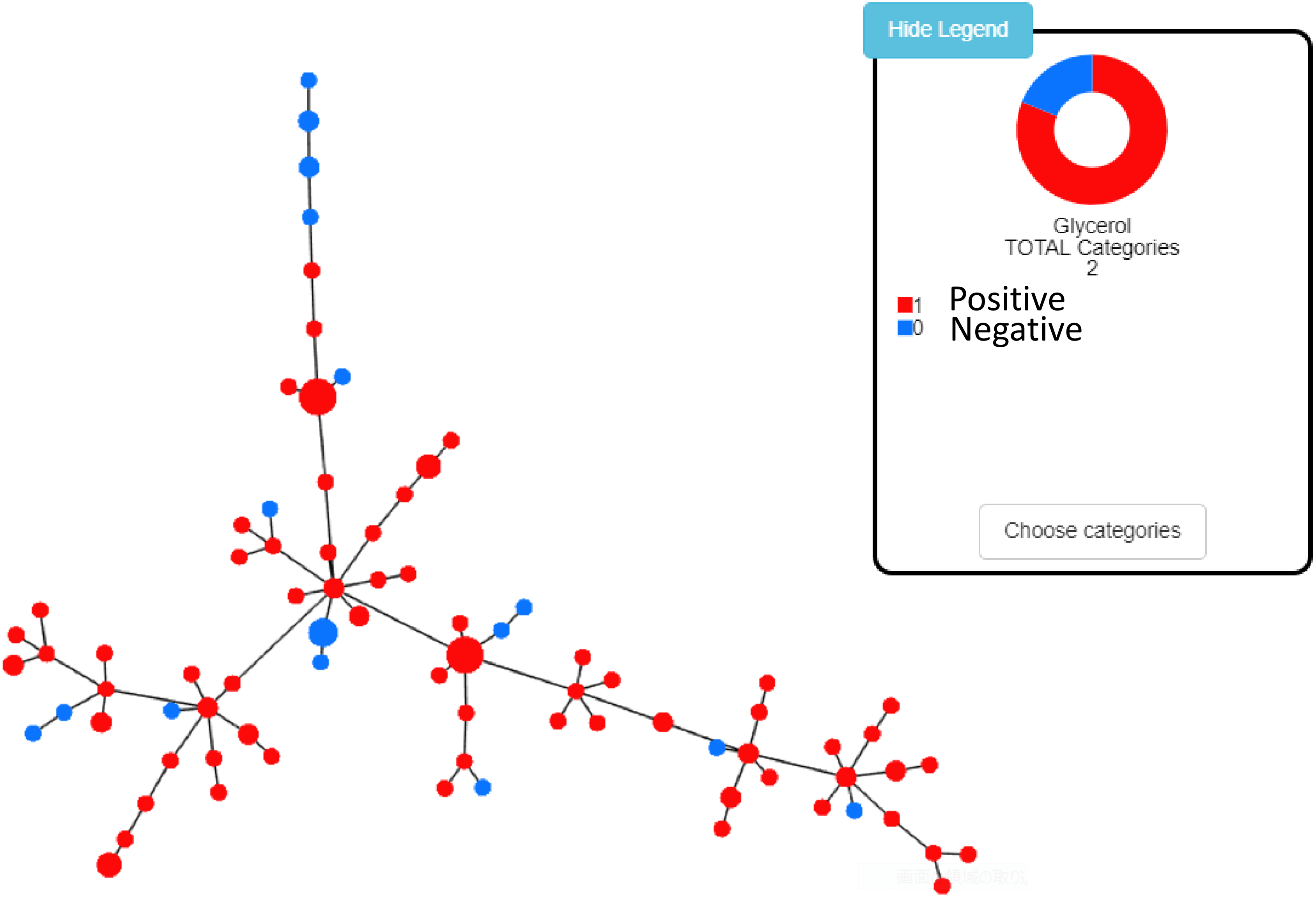

Glycerol #13 in Table 2

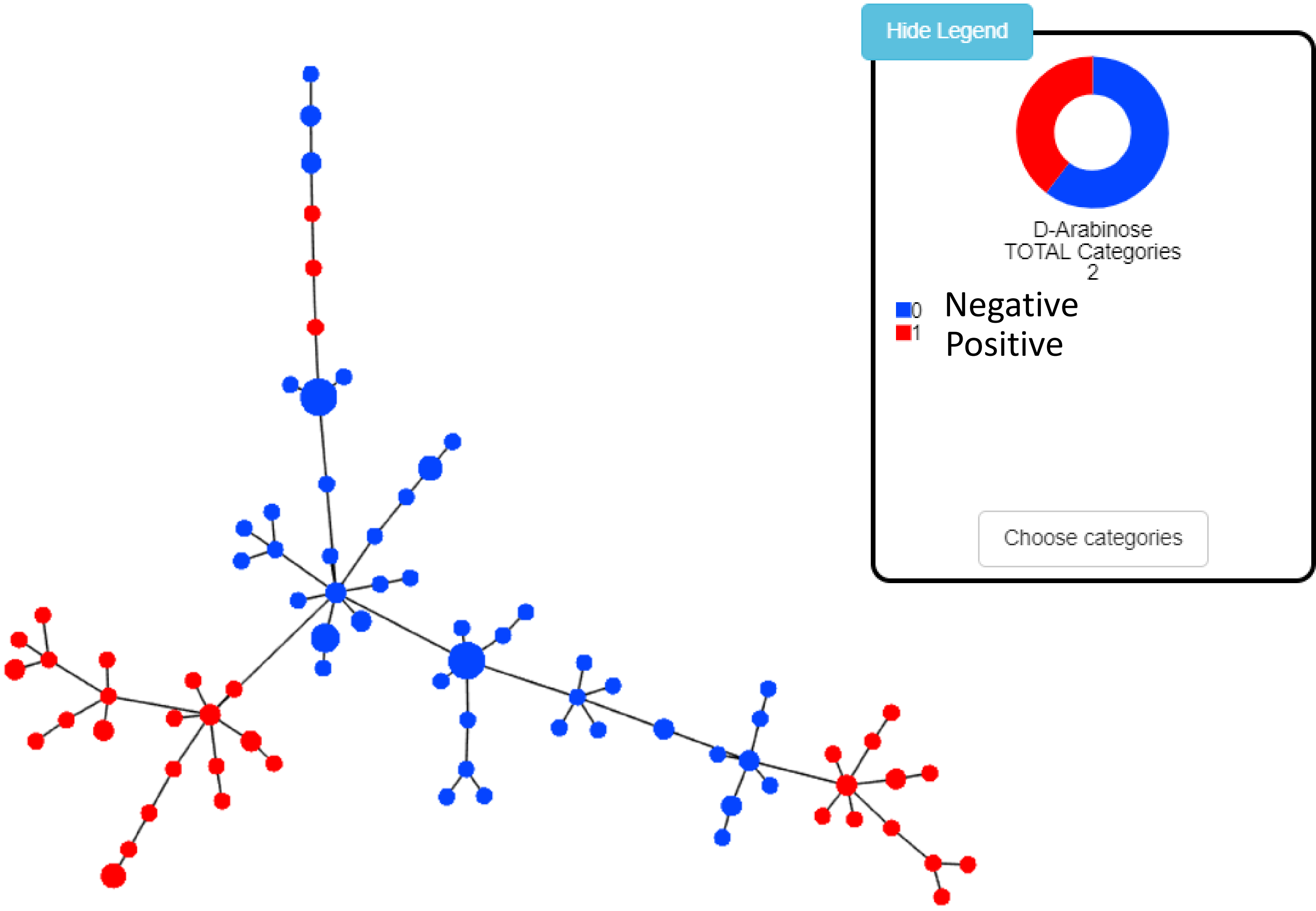

D-Arabinose #15 in Table 2

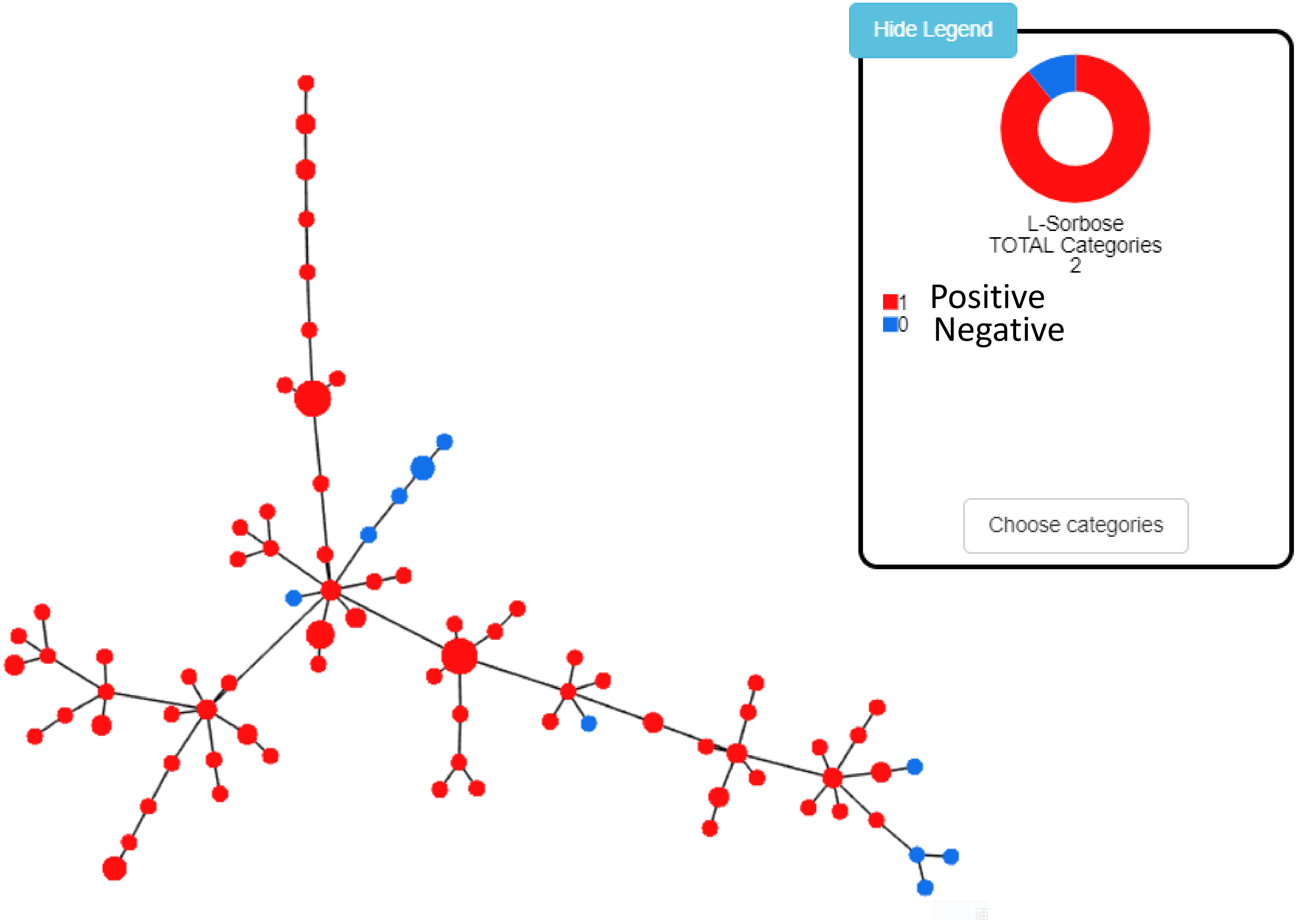

L-Sorbose #26 in Table 2

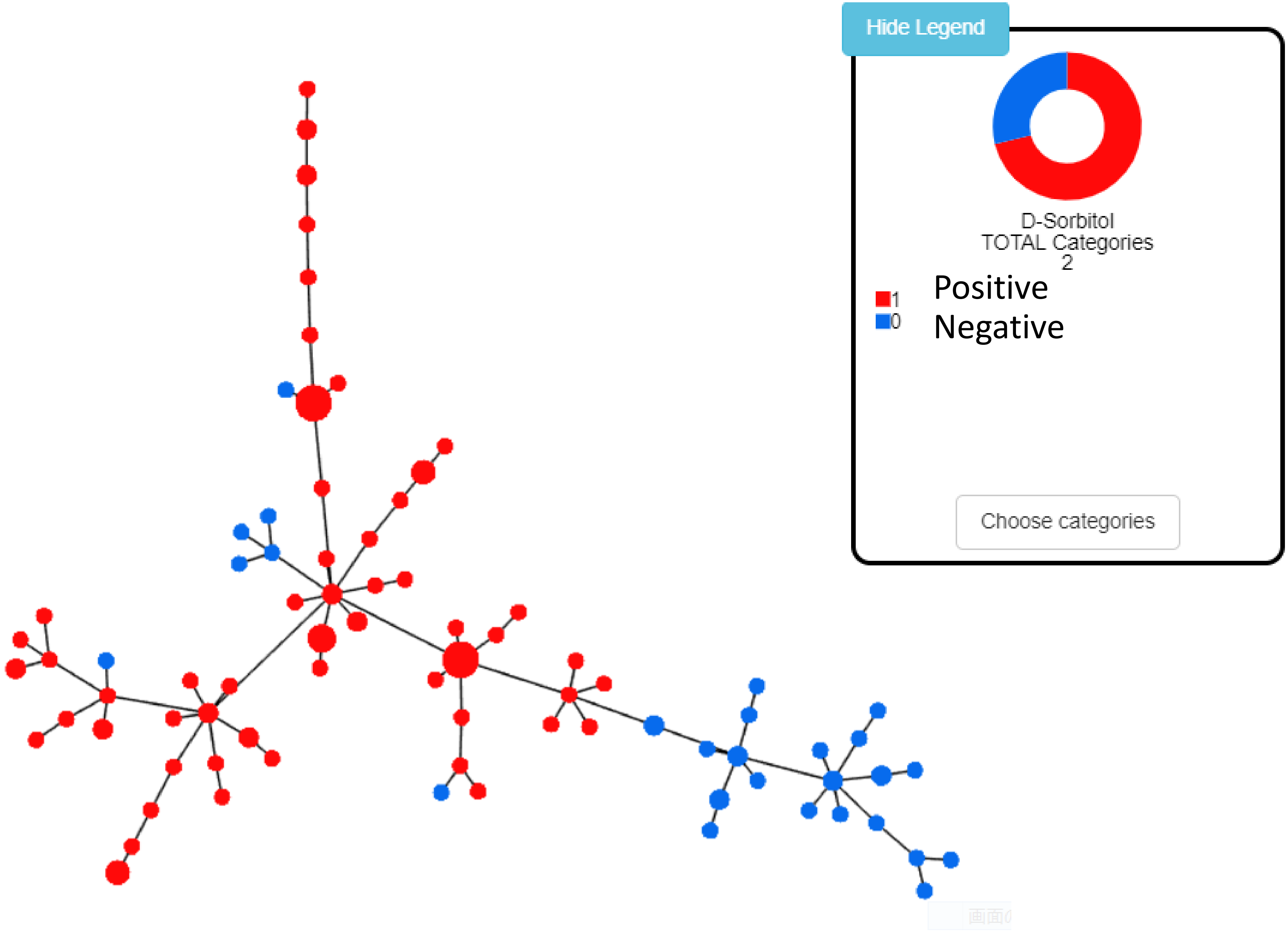

D-Sorbitol   #31 in Table 2

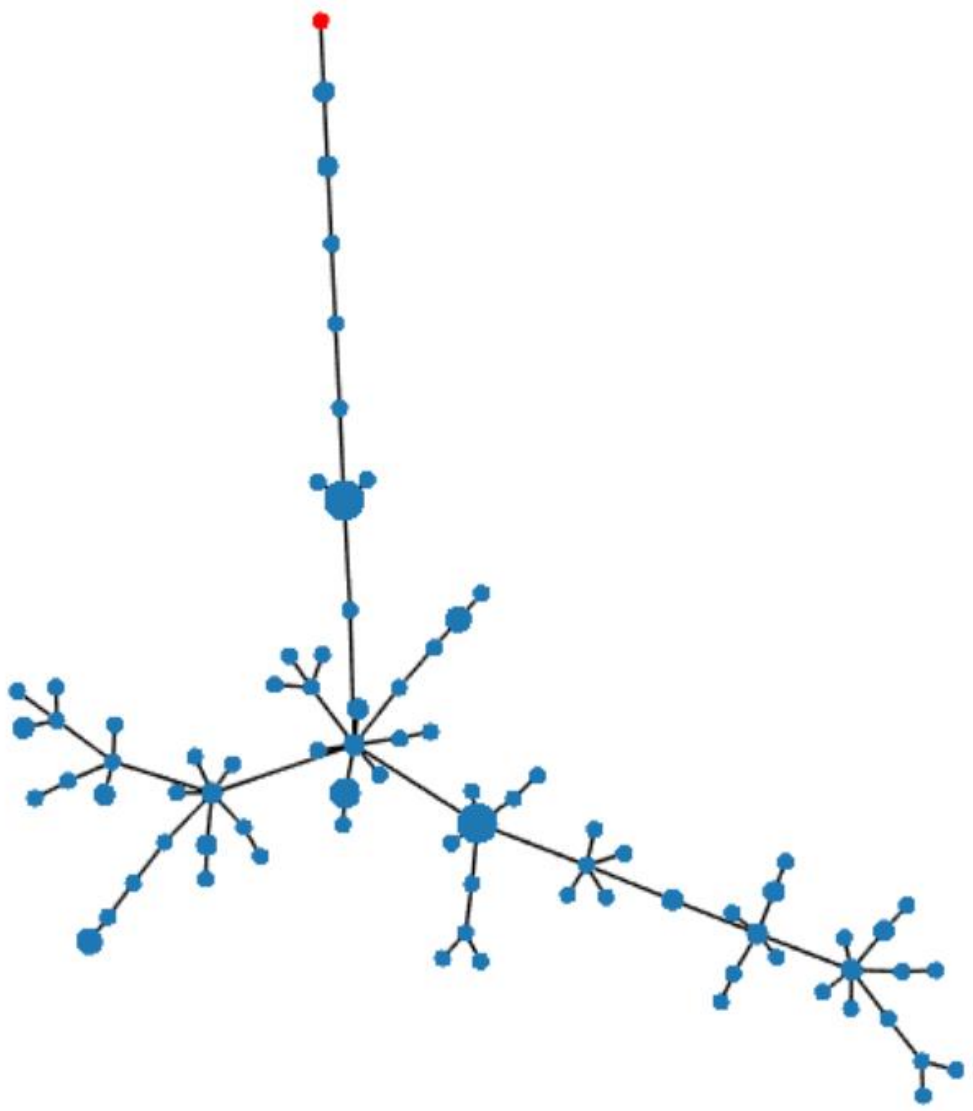

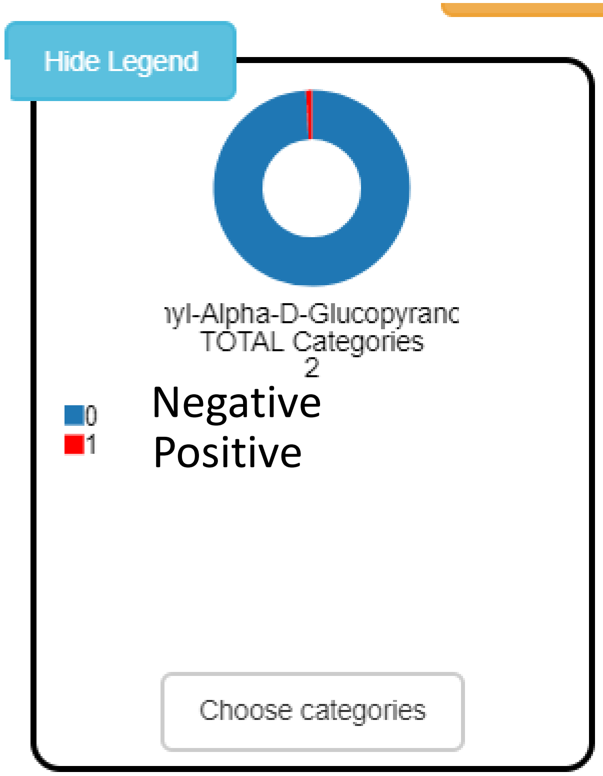

Methyl-α-D-glucopyranoside #33 in Table 2

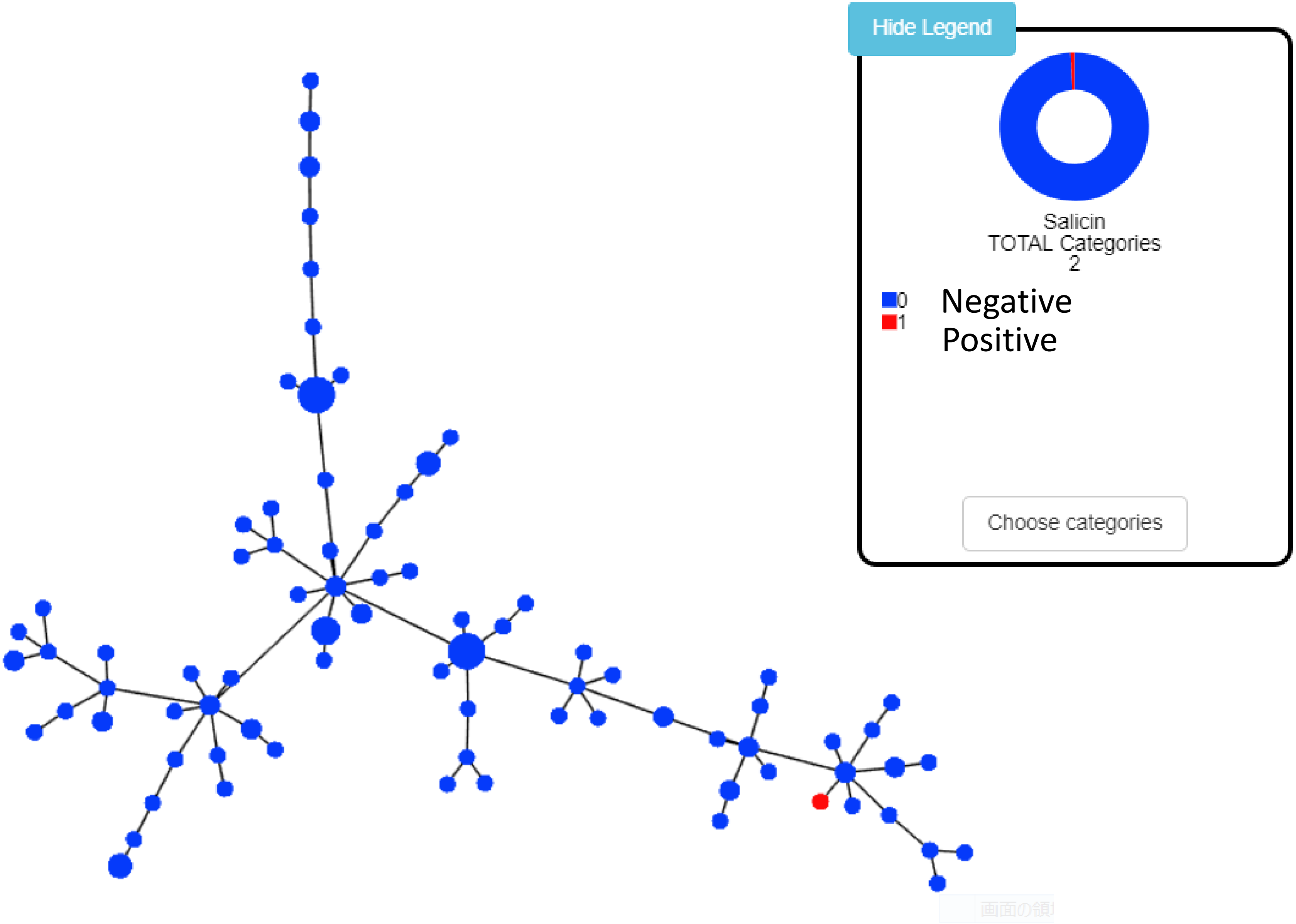

Salicin #38 in Table 2

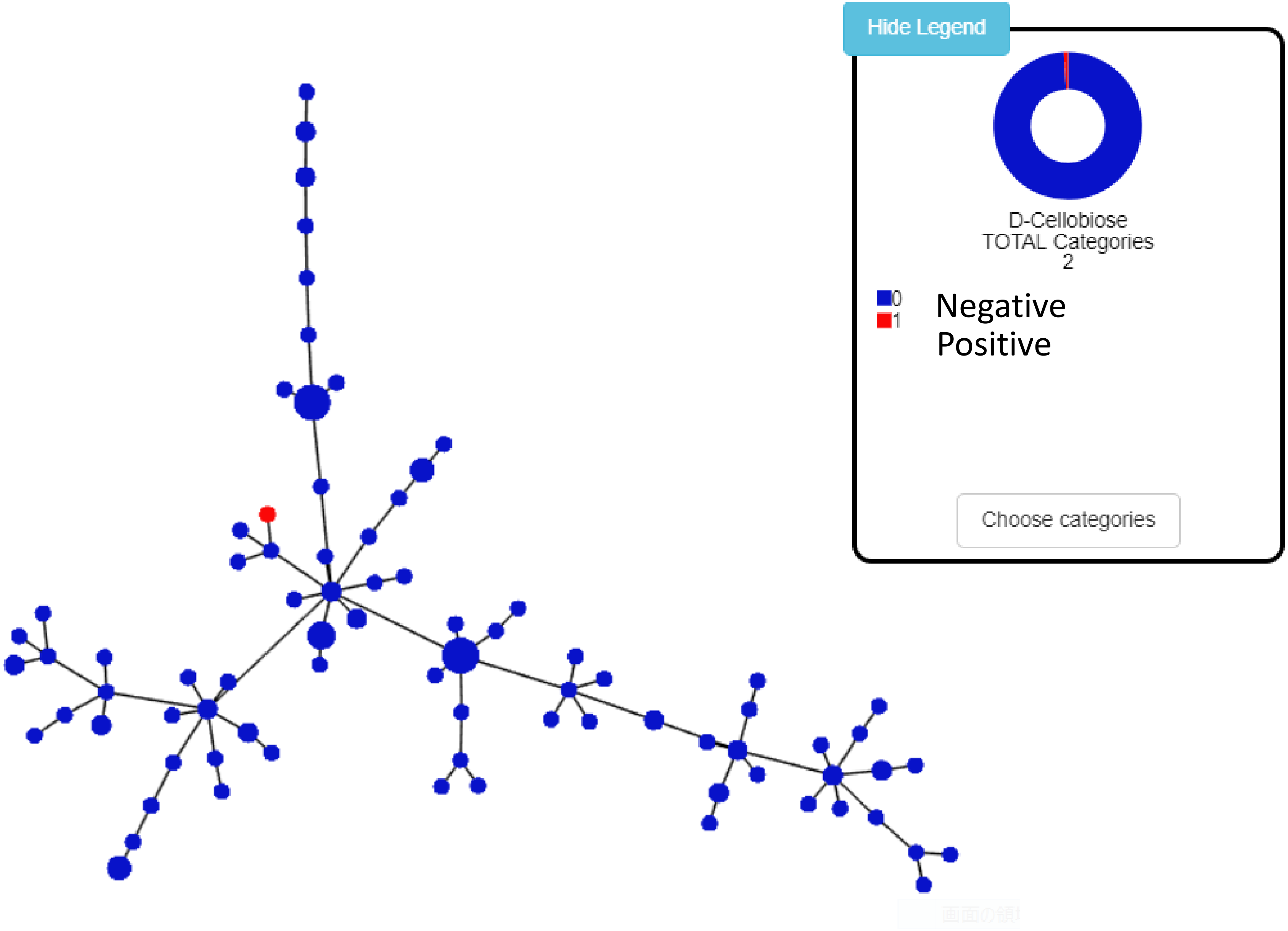

D-Cellobiose #39 in Table 2

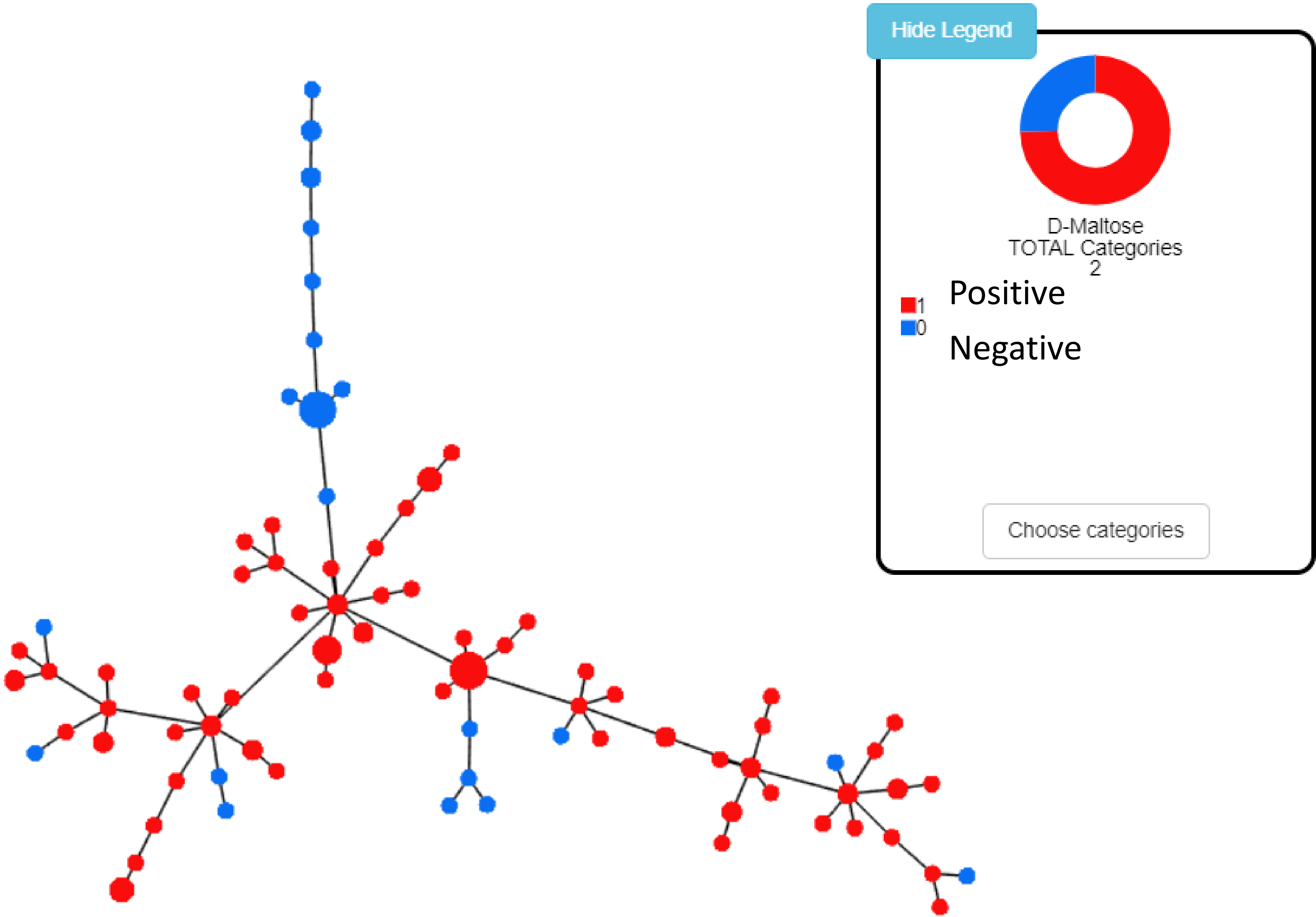

D-Maltose #40 in Table 2

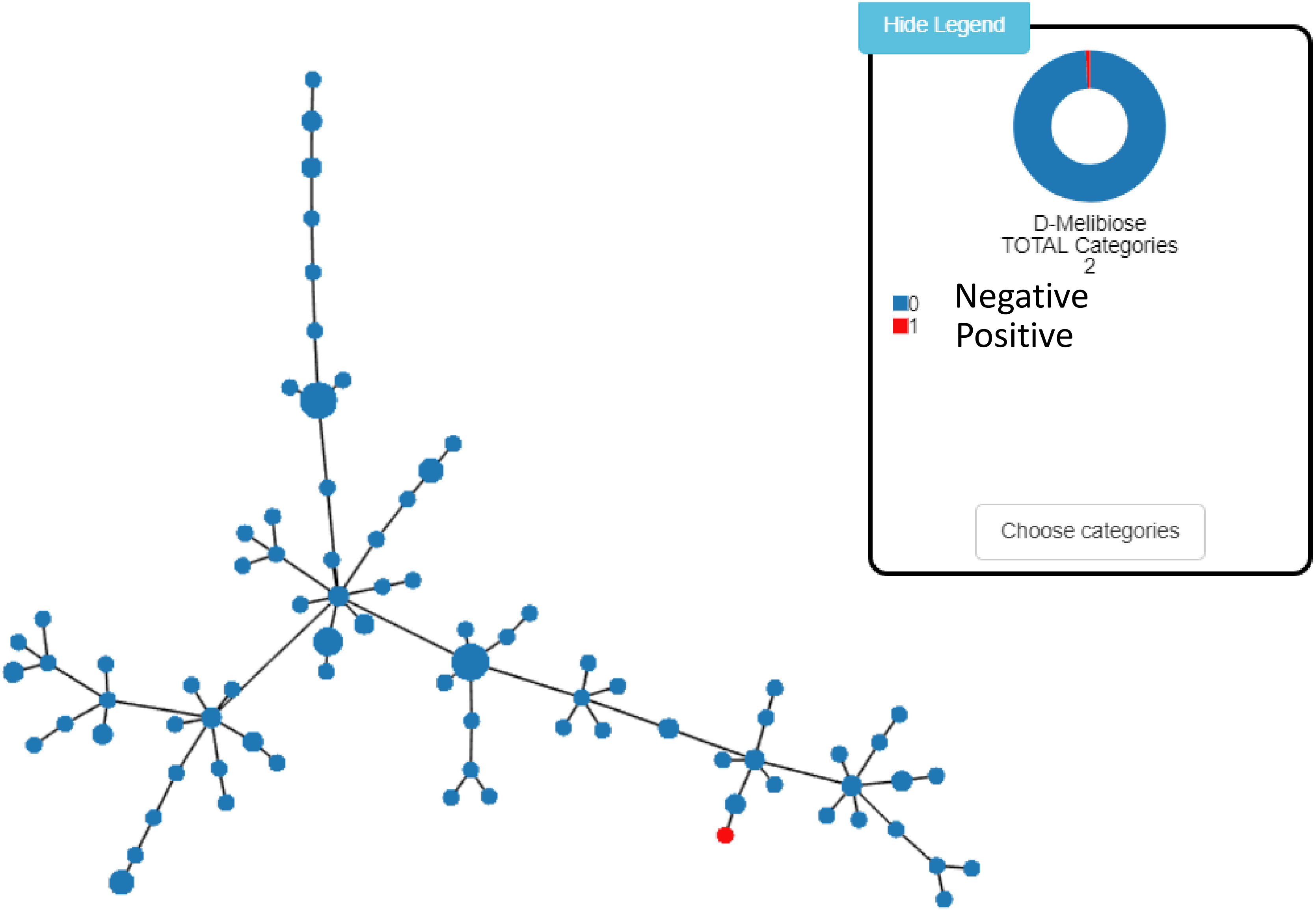

D-Melibiose #42 in Table 2

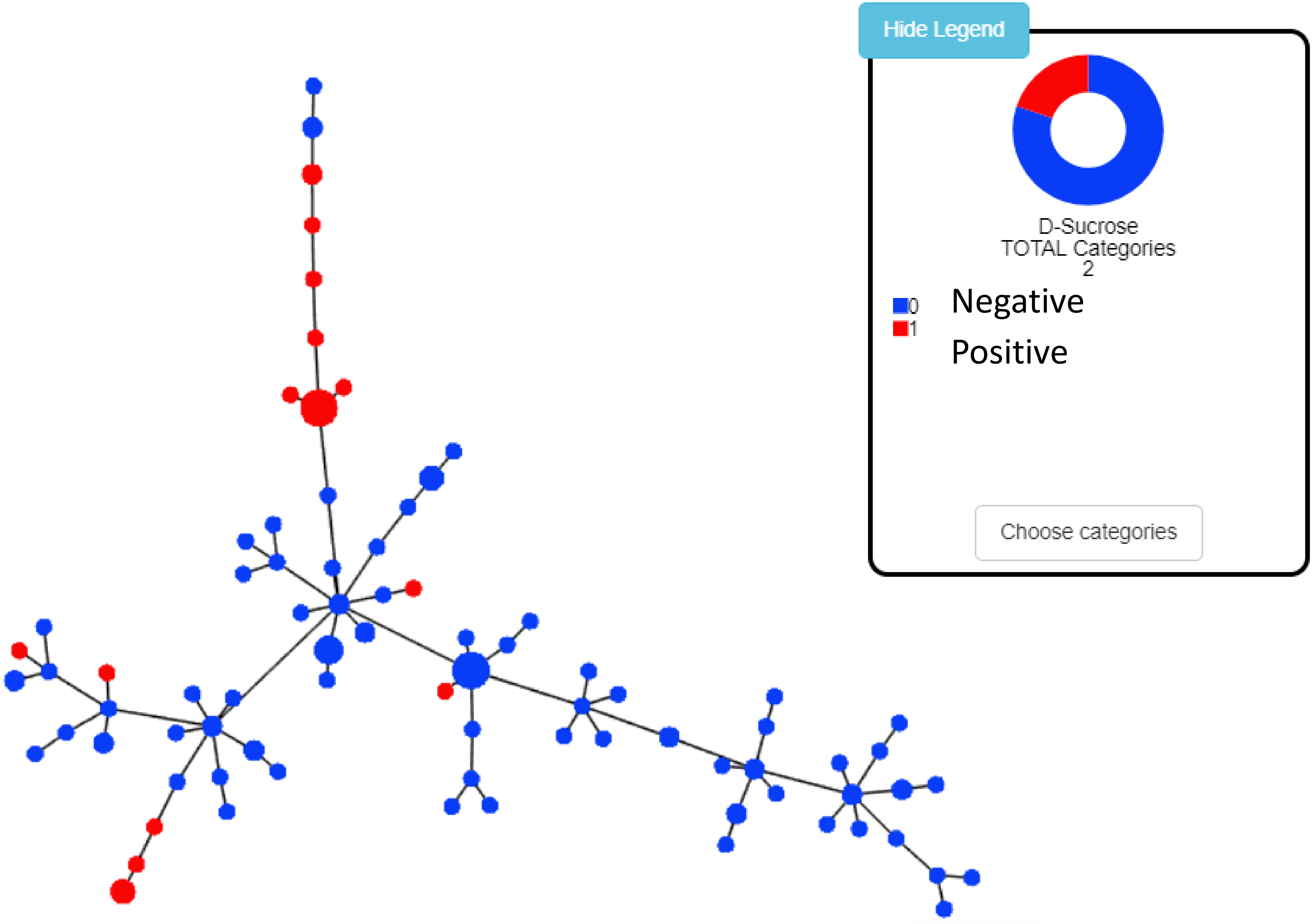

D-Sucrose #43 in Table 2

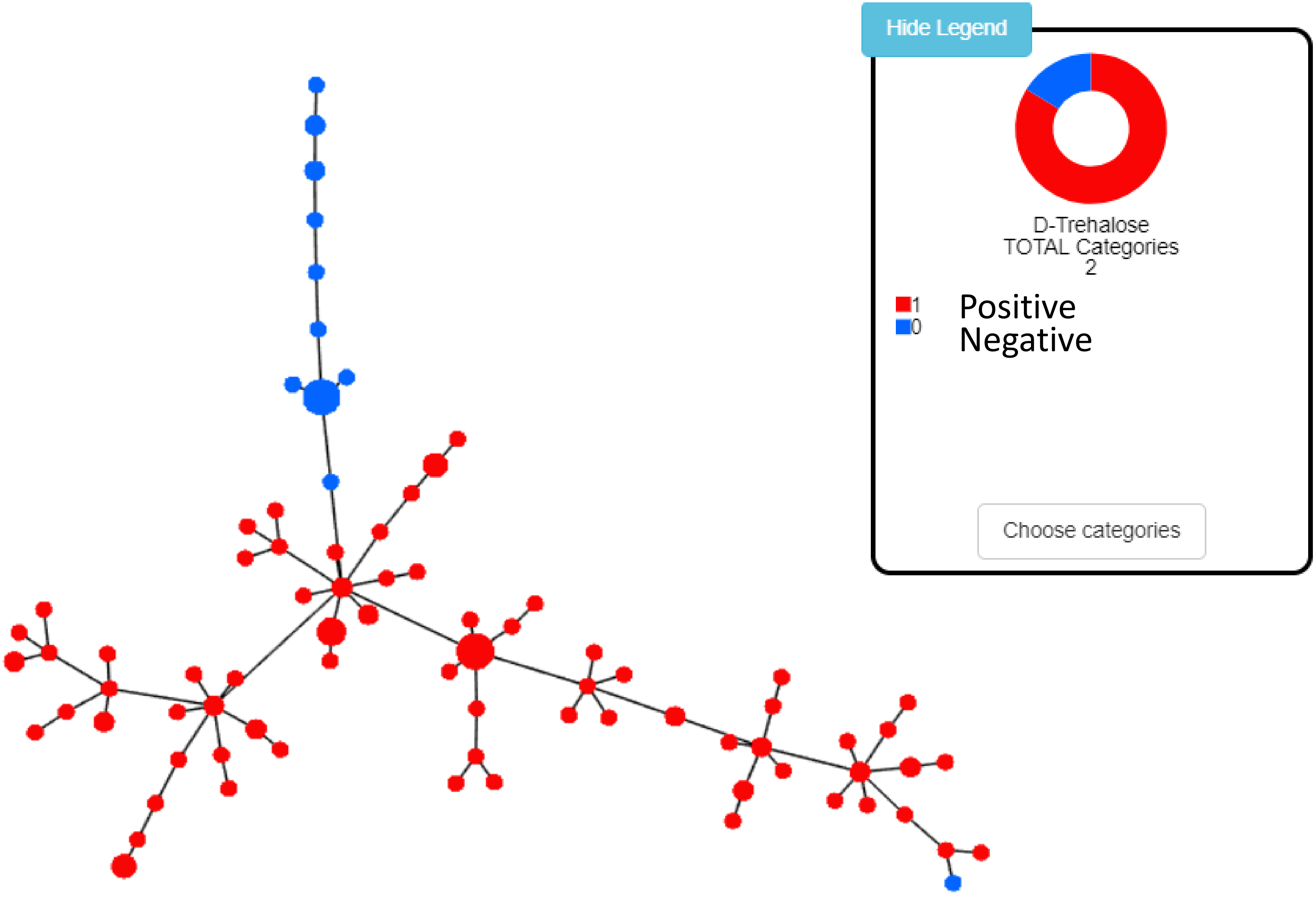

D-Trehalose #44 in Table 2

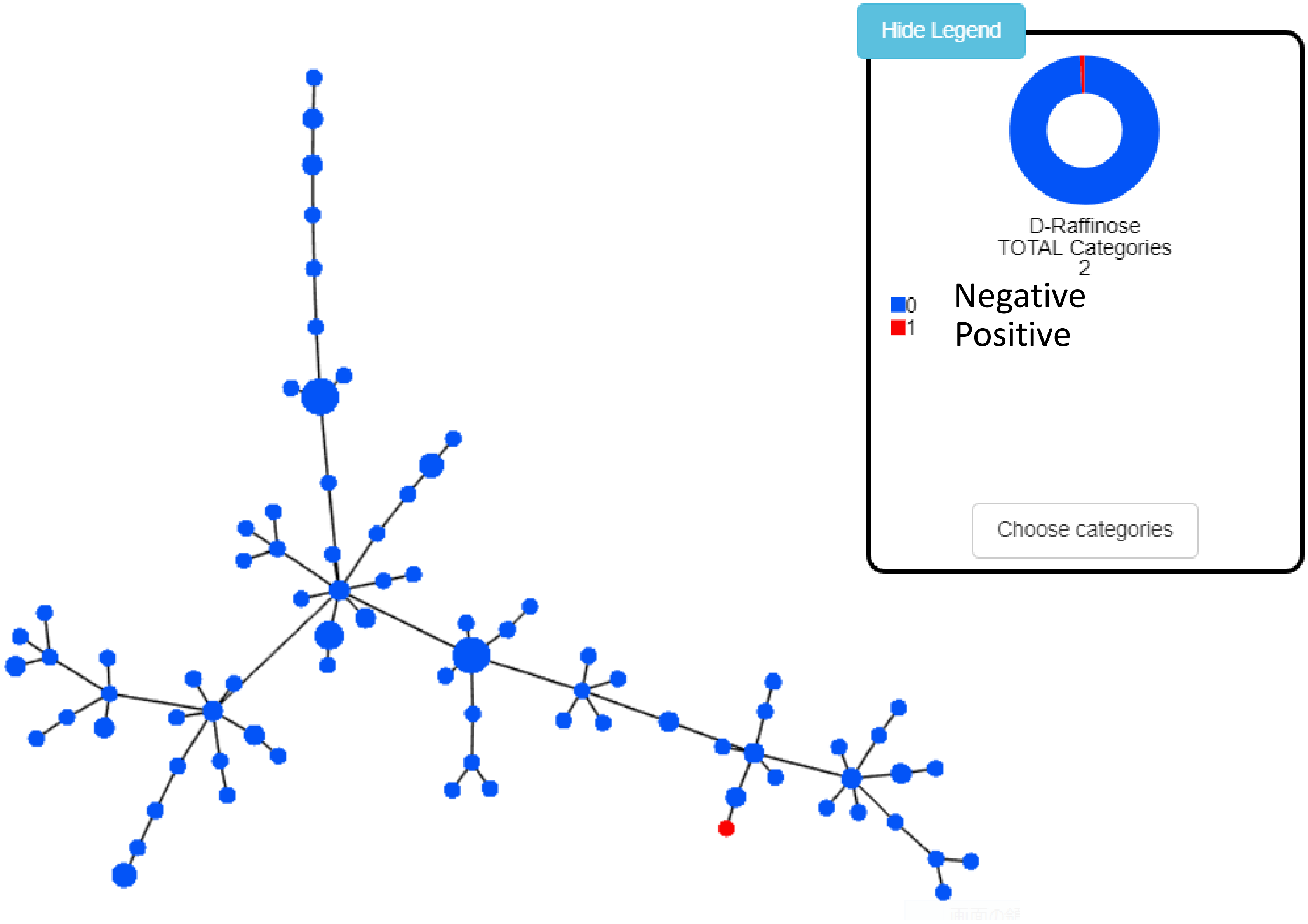

D-Raffinose

#47 in Table 2

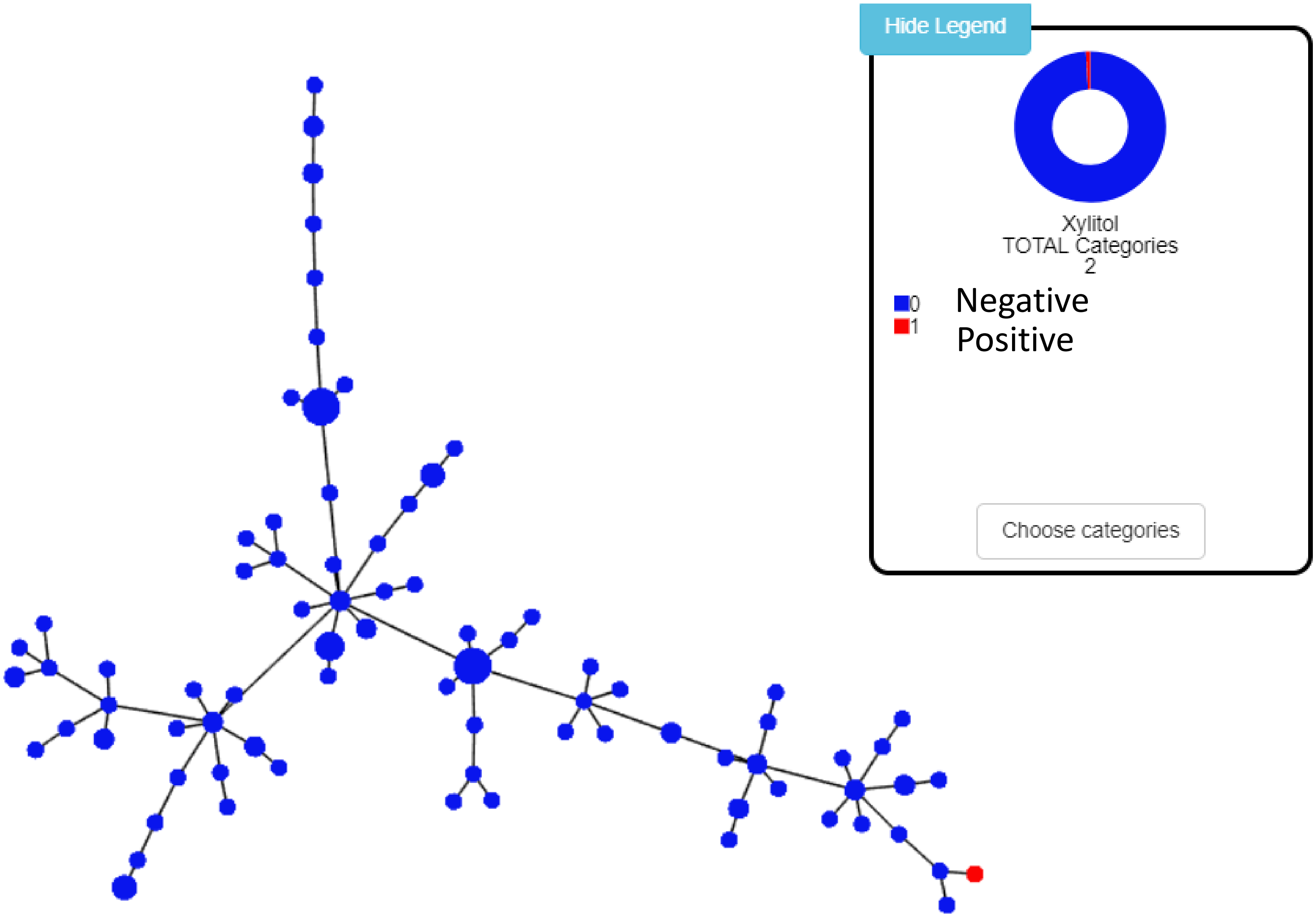

Xylitol #50 in Table 2

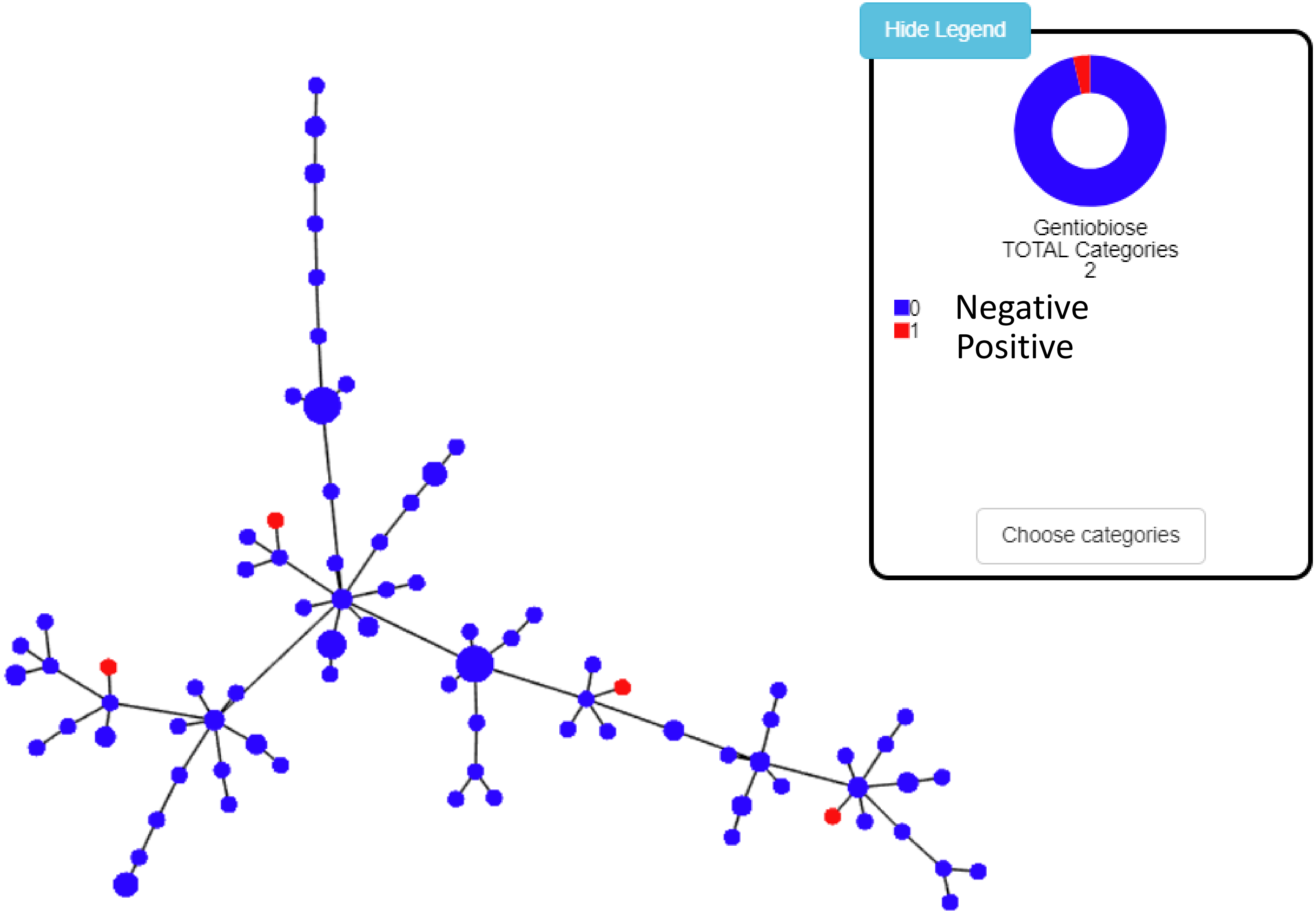

Gentiobiose #51 in Table 2

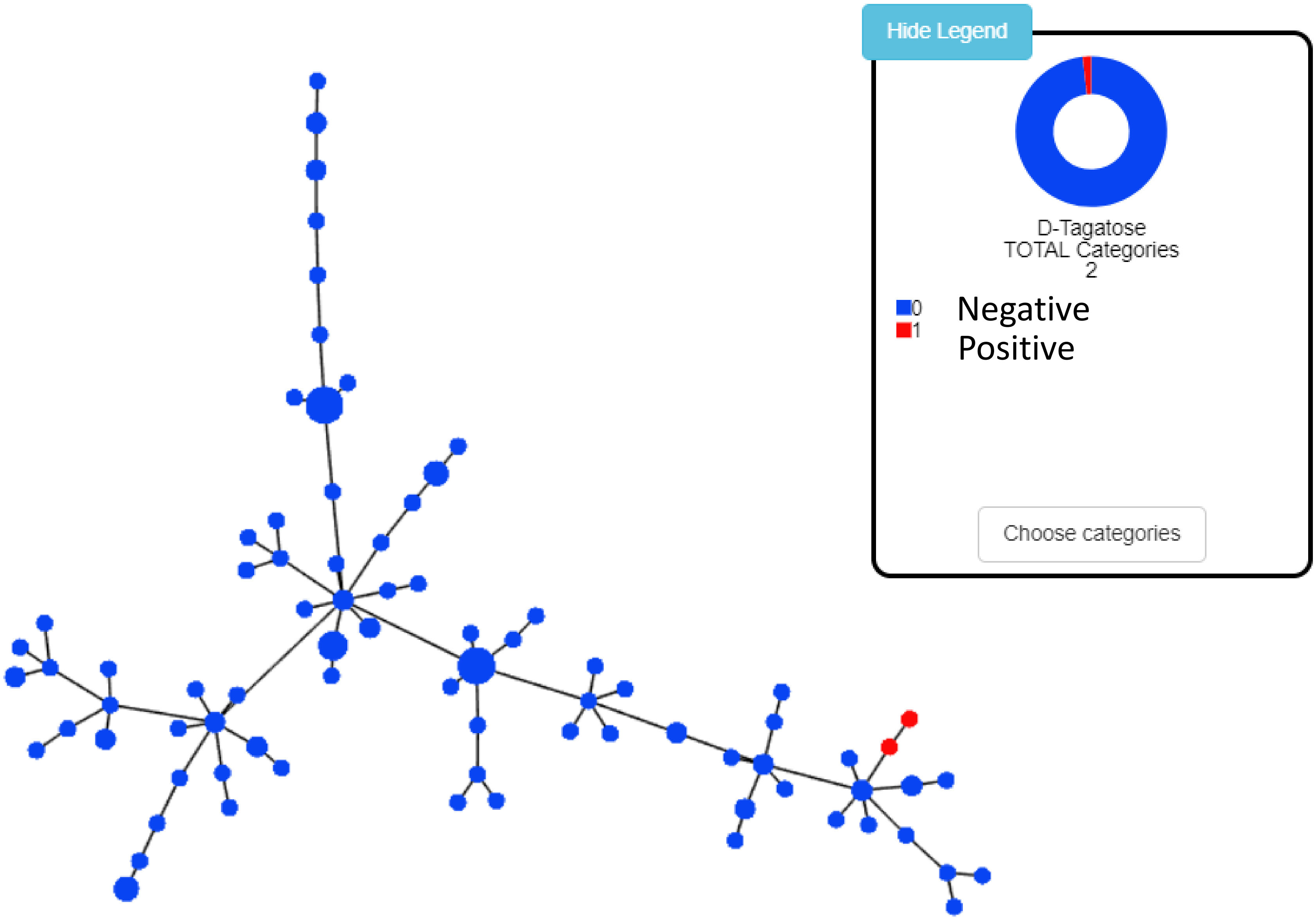

D-Tagatose #54 in Table 2

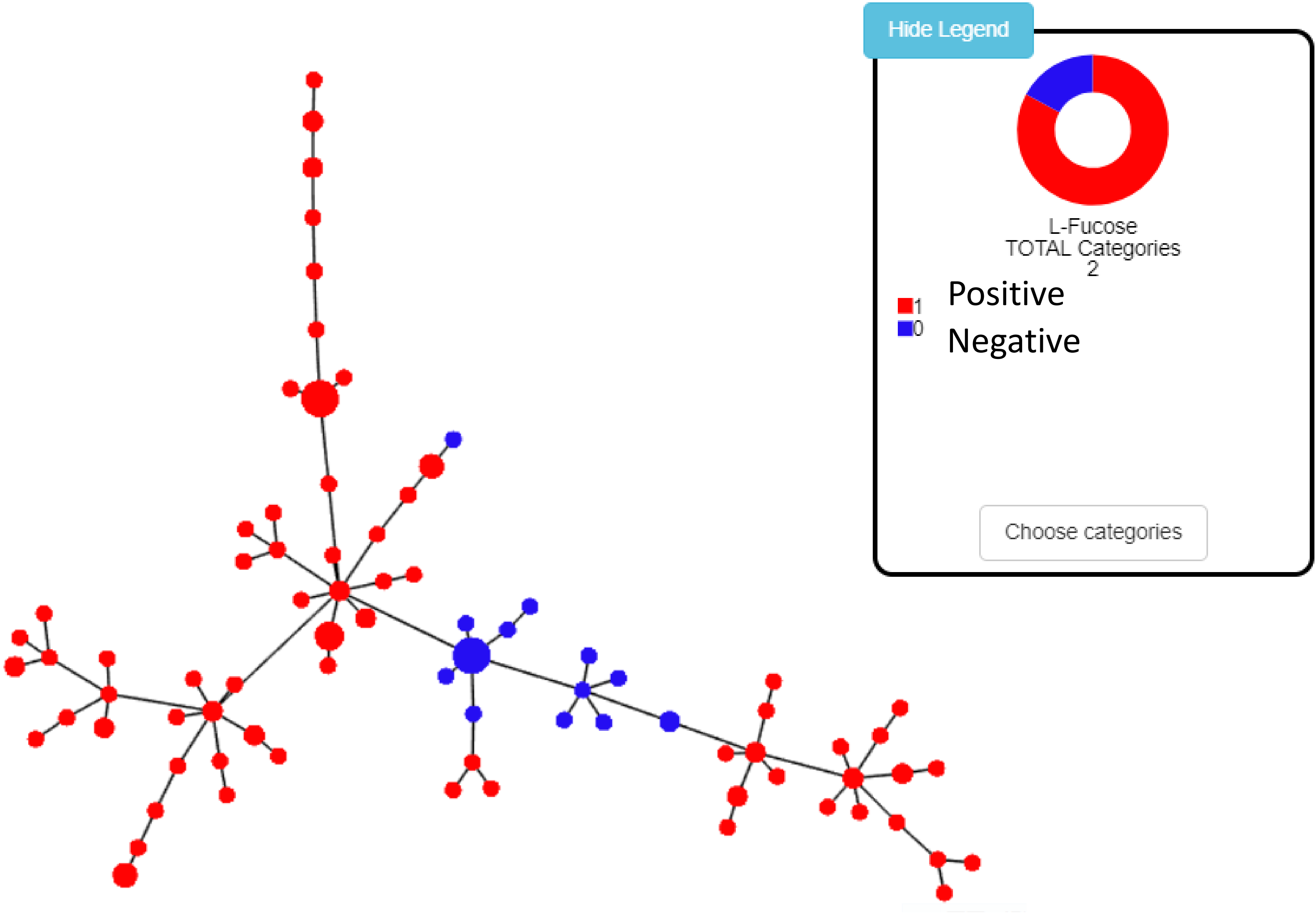

L-Fucose #56 in Table 2

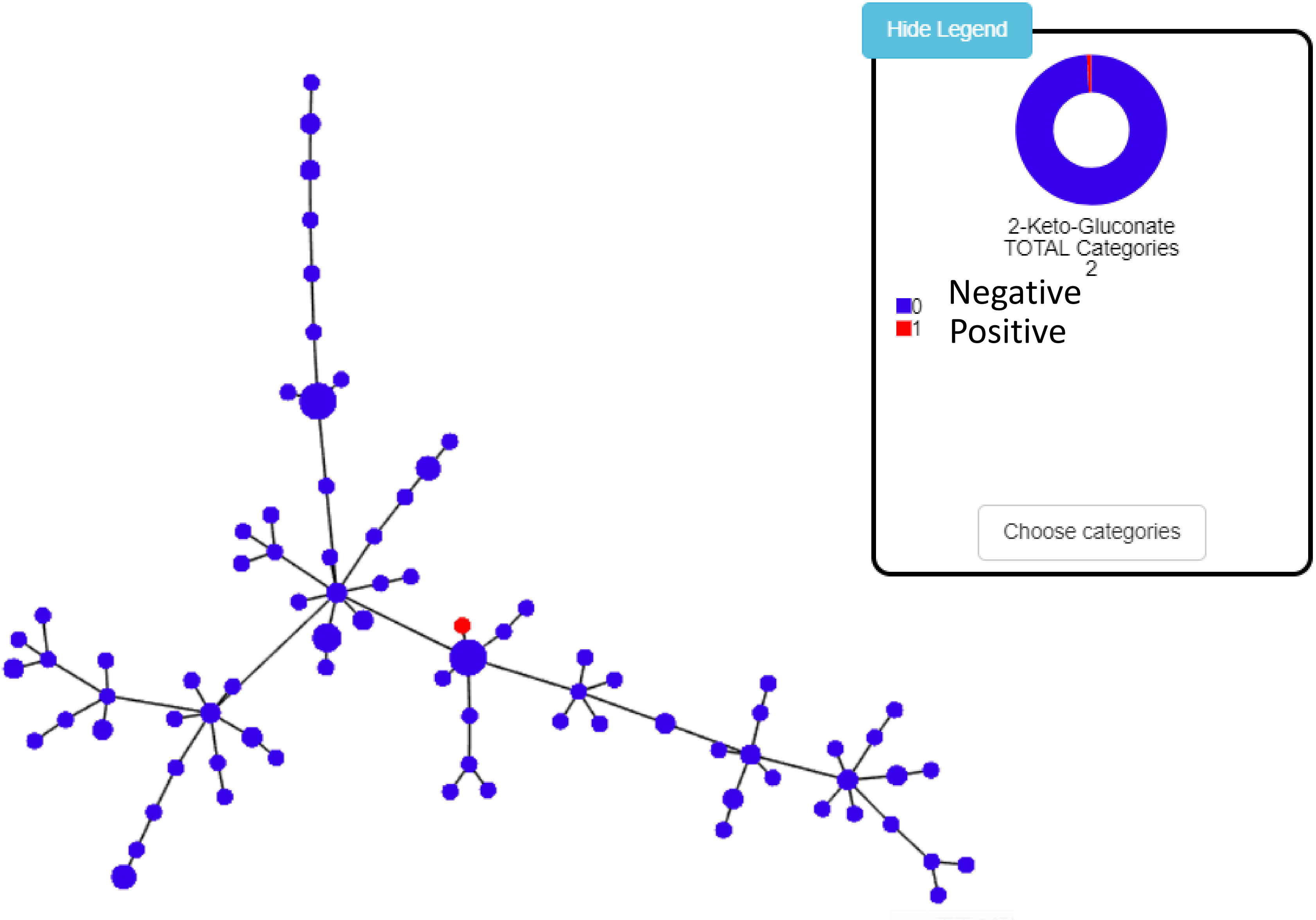

2-Keto-Gluconate #60 in Table 2

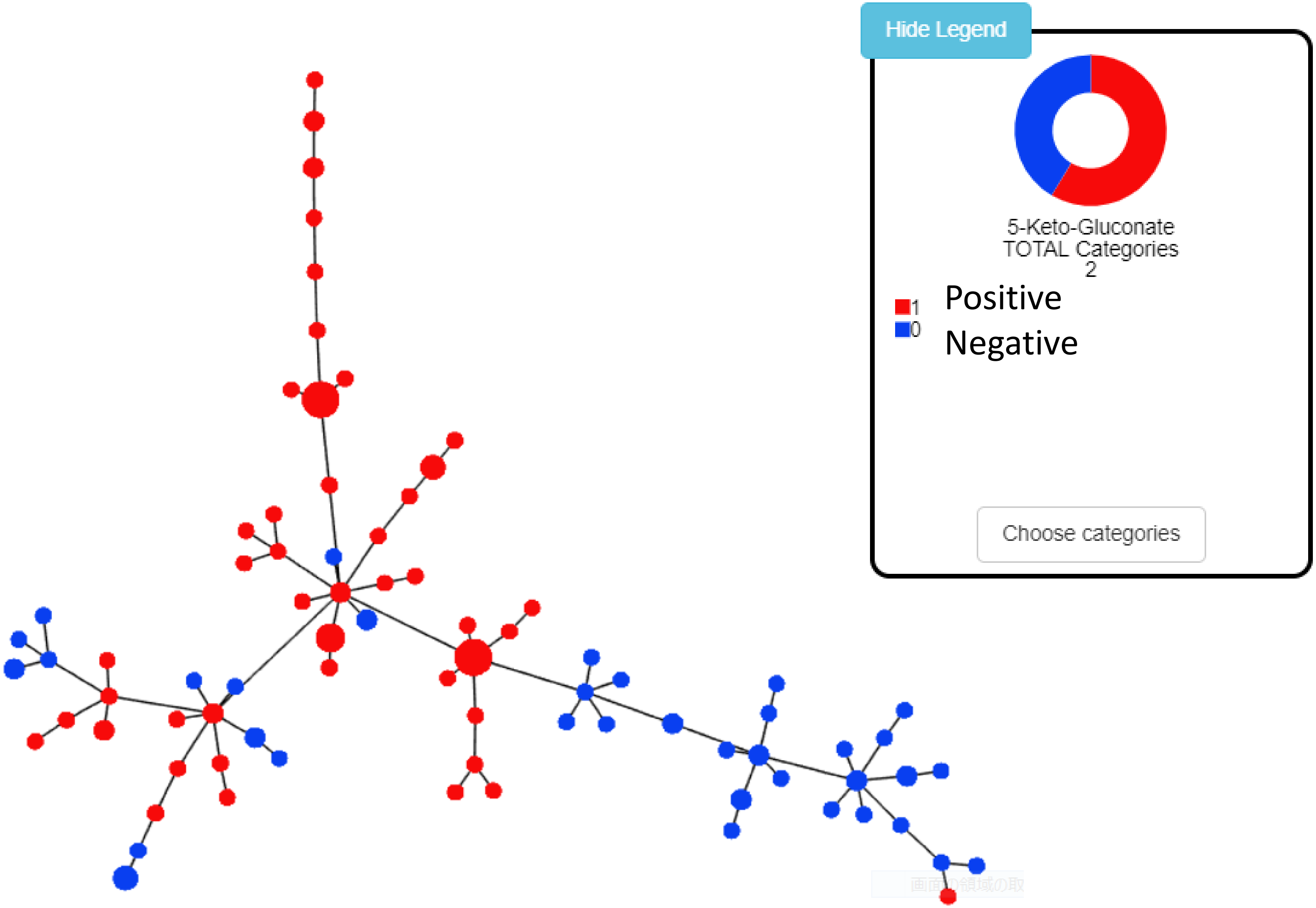

5-Keto-Gluconate #61 in Table 2

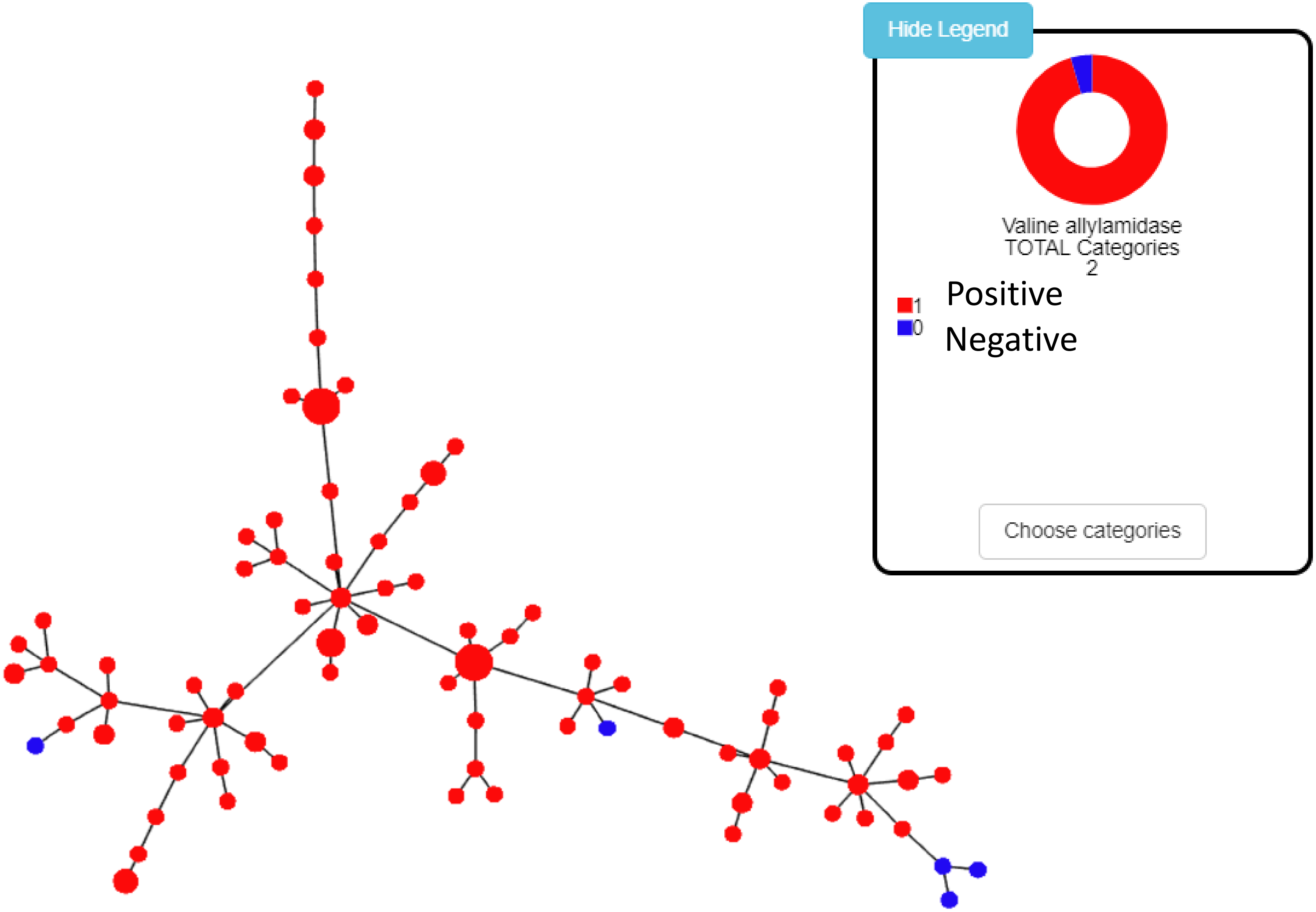

Valine allylamidase #67 in Table 2

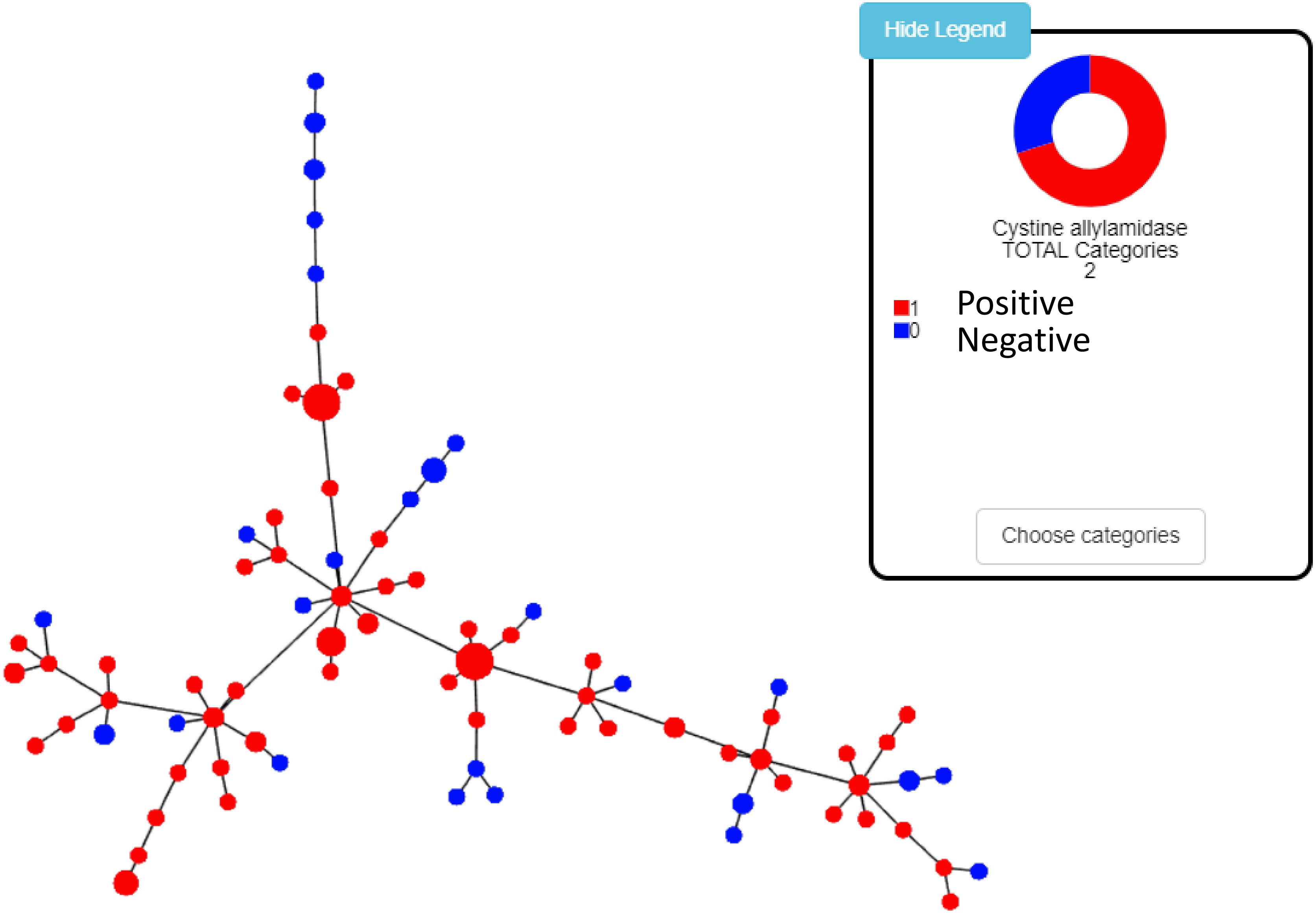

Cystine allylamidase #68 in Table 2

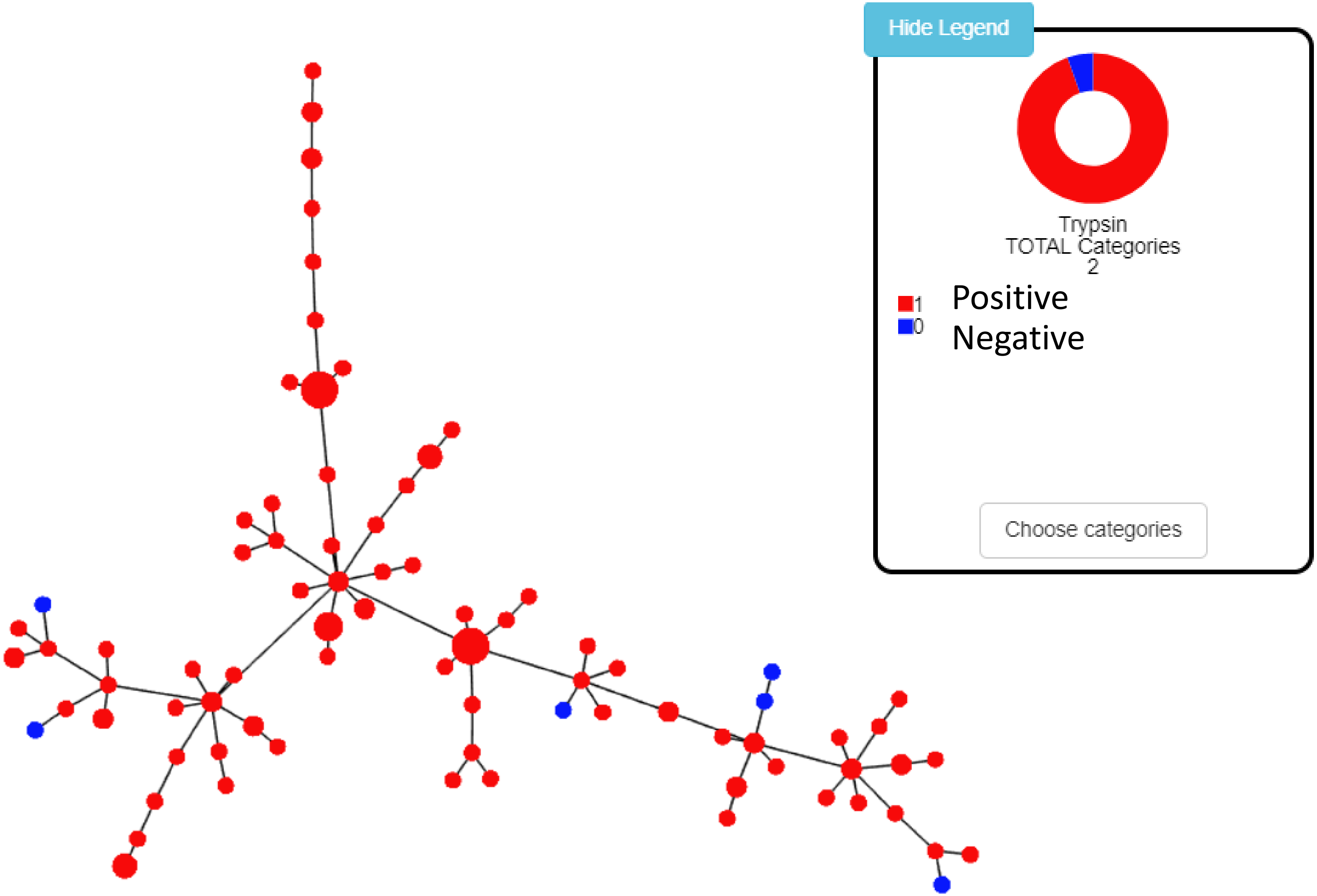

Trypsin

#69 in Table 2

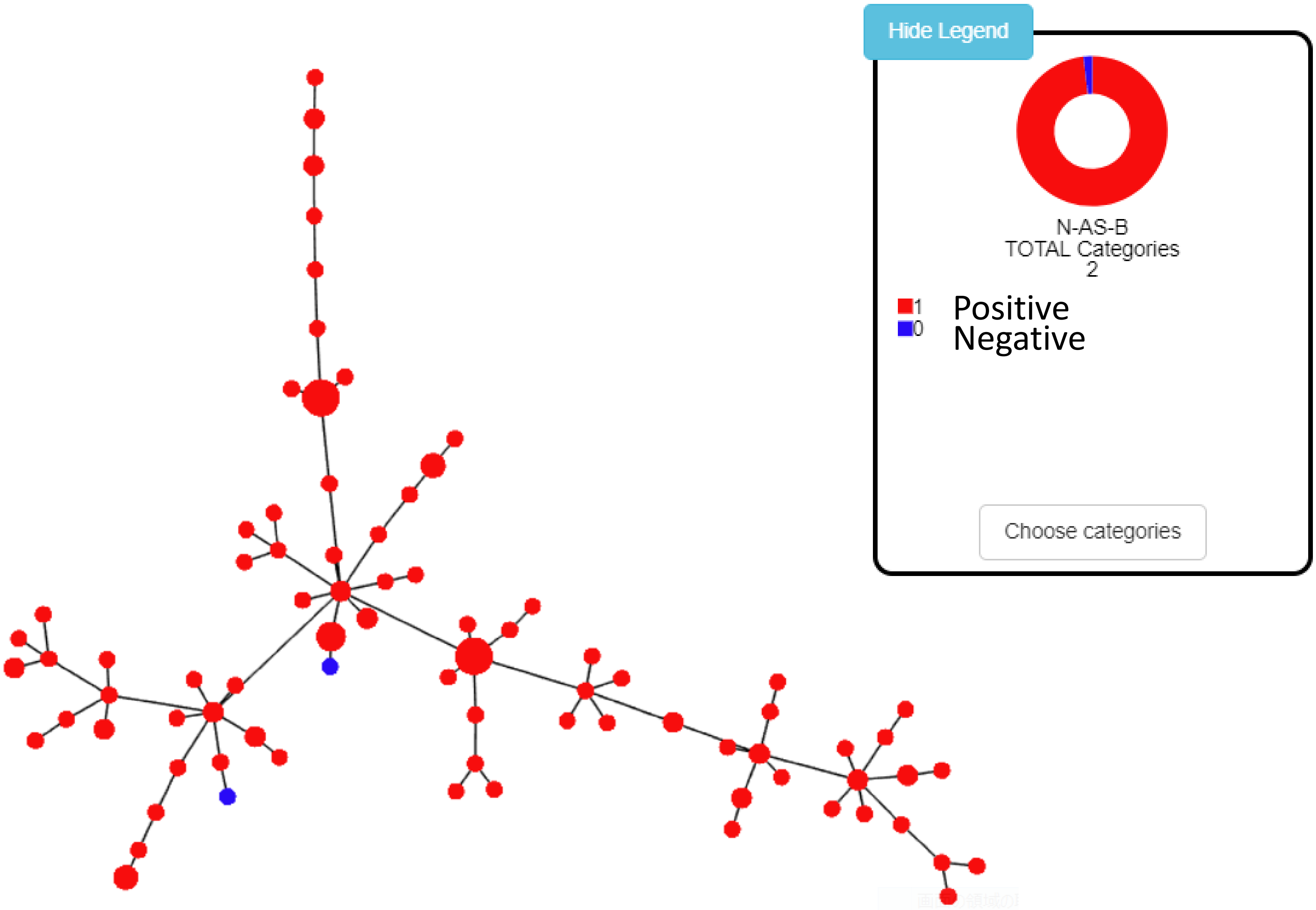

Naphthol-AS-BI-phosphohydrolase #72 in Table 2
